# Socioeconomic, Ethnocultural, Substance- and Cannabinoid-Related Epidemiology of Down Syndrome USA 1986–2016: Combined Geotemporospatial and Causal Inference Investigation

**DOI:** 10.3390/ijerph192013340

**Published:** 2022-10-16

**Authors:** Albert Stuart Reece, Gary Kenneth Hulse

**Affiliations:** 1 Division of Psychiatry, University of Western Australia, Crawley, WA 6009, Australia; 2 School of Medical and Health Sciences, Edith Cowan University, Joondalup, WA 6027, Australia

**Keywords:** cannabis, cannabinoid, Δ9-tetrahydrocannabinol, cannabigerol, cannabidiol, other drugs, socioeconomic, ethnocultural, down syndrome

## Abstract

Background: Down syndrome (DS) is the commonest of the congenital genetic defects whose incidence has been rising in recent years for unknown reasons. This study aims to assess the impact of substance and cannabinoid use on the DS Rate (DSR) and assess their possible causal involvement. Methods: An observational population-based epidemiological study 1986–2016 was performed utilizing geotemporospatial and causal inferential analysis. Participants included all patients diagnosed with DS and reported to state based registries with data obtained from National Birth Defects Prevention Network of Centers for Disease Control. Drug exposure data was from the National Survey of Drug Use and Health (NSDUH) a nationally representative sample interviewing 67,000 participants annually. Drug exposures assessed were: cigarette consumption, alcohol abuse, analgesic/opioid abuse, cocaine use and last month cannabis use. Covariates included ethnicity and median household income from US Census Bureau; maternal age of childbearing from CDC births registries; and cannabinoid concentrations from Drug Enforcement Agency. Results: NSDUH reports 74.1% response rate. Other data was population-wide. DSR was noted to rise over time and with cannabis use and cannabis-use quintile. In the optimal geospatial model lagged to four years terms including Δ9-tetrahydrocannabinol and cannabigerol were significant (from β-est. = 4189.96 (95%C.I. 1924.74, 6455.17), *p* = 2.9 × 10^−4^). Ethnicity, income, and maternal age covariates were not significant. DSR in states where cannabis was not illegal was higher than elsewhere (β-est. = 2.160 (1.5, 2.82), R.R. = 1.81 (1.51, 2.16), *p* = 4.7 × 10^−10^). In inverse probability-weighted mixed models terms including cannabinoids were significant (from β-estimate = 18.82 (16.82, 20.82), *p* < 0.0001). 62 E-value estimates ranged to infinity with median values of 303.98 (IQR 2.50, 2.75 × 10^7^) and 95% lower bounds ranged to 1.1 × 10^71^ with median values of 10.92 (IQR 1.82, 7990). Conclusions. Data show that the association between DSR and substance- and cannabinoid- exposure is robust to multivariable geotemporospatial adjustment, implicate particularly cannabigerol and Δ9-tetrahydrocannabinol, and fulfil quantitative epidemiological criteria for causality. Nevertheless, detailed experimental studies would be required to formally demonstrate causality. Cannabis legalization was associated with elevated DSR’s at both bivariate and multivariable analysis. Findings are consistent with those from Hawaii, Colorado, Canada, Australia and Europe and concordant with several cellular mechanisms. Given that the cannabis industry is presently in a rapid growth-commercialization phase the present findings linking cannabis use with megabase scale genotoxicity suggest unrecognized DS risk factors, are of public health importance and suggest that re-focussing the cannabis debate on multigenerational health concerns is prudent.

## 1. Introduction

Down syndrome (DS) was first described by British physician John Down in 1866 [1] and is well known to include a variety of facial features, mild to moderate growth retardation, mild to moderately impaired intellectual development, a single palmar crease, loose muscle tone or joints and a curved little finger [2]. It also includes a number of lesser known features including reduced life expectancy, a 6% rate of myeloid leukaemia development and a number of increased co-morbidities including congenital heart disease [2,3,4]. As it was shown in 1959 that the cause of the syndrome is an extra chromosome 21 [1] it represents an example of a disorder with an altered genome. Down syndrome is increasingly common which is usually attributed to women having children later in life. Indeed the only cause mentioned on the Centres for Disease Control (CDC) website relating to this disorder is advanced maternal age [2]. However, society is changing in other ways, particularly in exposure to drug substances. US data show that the overall use of tobacco and alcohol products is declining [5]. In some nations opioid abuse has become widespread and the cannabis industry appears to be entering a rapidly developing commercialization phase in nations such as Canada and USA. 

We were particularly intrigued by the demonstration in Hawaii in 2007 that cannabis use alone was associated with an elevated risk of Down syndrome with a report of a rate ratio of 5.26 (95%C.I. 1.08–15.46) on the basis of only 3 exposed cases amongst 479 total cases [6]. Researchers from Canada Health have also reported higher rates of Down syndrome in the northern territories of Canada where more cannabis is known to be consumed [7,8,9,10]. A similar link was recently shown in Colorado where Down syndrome has risen over the decade of cannabis legalization and where the rise is associated more with increased cannabis use during a period where rates of tobacco and alcohol consumption were declining [11,12]. Similar findings also emerge from an Australian report [13] and recent European reports [14,15,16]. Interestingly congenital heart disease has also increased in line with cannabis consumption in Hawaii, Canada, Colorado and Europe [6,8,12,14,15,17,18,19]. However, the important issue of the relationship of cannabis use with Down syndrome in the rich databases of the USA more generally has not as yet been considered in detail but has only been mentioned *en passant* [20]. We chose to focus on cannabis use since whilst similar associations have been described for cannabis they have not been described for other drugs.

Recent reports have found strong bivariate relationships between exposure to cannabis and many cannabinoids and both the raw Down syndrome rate (DSR) and estimates of the DSR corrected for early termination of pregnancy for anomaly (ETOPFA) across USA [17]. Similar recent powerful reports have issued from Europe [14,15]. However, this link has not been studied in a formal space-time relationship within a causal inferential framework in USA. The present report addresses this knowledge gap.

We therefore formed three related hypotheses prior to commencing our study. The first was that a demographic survey of drug use would be highly correlated with DS rates (DSR) across the USA after controlling for common covariates such as ethnicity, median household income and maternal age. Our second hypothesis was that cannabis use or cannabinoid exposure would be positively correlated with DSR epidemiologically across both space and time. The third hypothesis was that the various legal paradigms relating to cannabis may be significantly related to the DSR.

## 2. Methods

### 2.1. Data 

Data on Down Syndrome rates was taken from the annual reports of the National Birth Defects Prevention Network (NBDPN) overseen by the CDC Atlanta Georgia 1986–2016 [21]. National US reports collate state based data which generally relate to five year periods, the latest being 2012–2016. Case ascertainment style for each registry was taken from the NBDPN annual reports. Drug exposure data was taken on a state basis from the National Survey of Drug Use and Health (NSDUH) an annual survey conducted by the Substance Abuse and Mental Health Services Administration (SAMHSA) which is a nationally representative survey of the non-institutionalized US population over 18 years [22]. The main drugs of interest and their NSDUH abbreviations were cigarette use in the past month (cigmon), abuse or dependence on alcohol in the past year (abodalc), last month cannabis use (mrjmon), past year analgesic abuse (anlyr) and past year cocaine use (cocyr). Birth census and maternal age structure data was obtained from the CDC Wonder birth registries [23]. National state ethnicity figures and median household income were downloaded from US Census Bureau via tidycensus in “R”. Cannabinoid concentration data was taken from published Drug Enforcement Agency reports [24,25].

### 2.2. Derived Data 

National cannabinoid concentration data was multiplied by state-based measures of monthly cannabis use to derive an estimate of state based exposure to the various cannabinoids. NSDUH data was used to derive a mean number of days of cannabis use by ethnicity at the national level. This was multiplied by the state monthly cannabis use and by the reported THC potency to derive an index of state-based ethnic exposure to Δ9-tetrahydrocannabinol (THC). States were divided into cannabis-use quintiles based on their ranking in the 2015 NSUDH survey. Similarly NSDUH data was used to calculate an average mean number of days of cannabis used in pregnancy nationally also. This was multiplied by mrjmon to derive a local estimate of state-based pregnancy use denoted “First Trimester Cannabis Exposure”. CDC birth census data was used to derive a state- and year- specific fraction of mothers giving birth who were over 35 years of age to account for the known age effect on DS incidence.

The only longitudinal time series which could be identified describing the time course of early termination of pregnancy for anomaly (ETOPFA) for DS was that from the Western Australia Registry of Developmental Anomalies (WARDA) 1980–2014 [26]. The present ETOPFA for Down syndrome is 70%. The time course of the increase from the WARDA dataset until present was used to calculate a fractional maximal ETOPFA rate (FMaxTR) and the raw NBDPN-reported rates were standardized against that to derive an annual estimate of DSR inclusive of ETOPFA’s. These data are supported by other international series [27,28,29]. 

### 2.3. Statistical Analysis and Data Cleaning 

Data was processed in “R-Studio” version 1.2.1335 based on “R” from CRAN version 3.6.1. Data was manipulated and matched in R packages base and dplyr [30], linear regression was performed in base, graphs were drawn in ggplot2 [30] and sf [31] and geofacetted in geofacet [32], correlation matrices were visualized in corrgram [33,34], panel regression was conducted in plm [35], robust sandwich regressoin was performed in survey [36], spatial weights and matrices were calculated in spdep [37], and spatial regression was performed in splm [38,39]. Variables were log transformed based on the Shapiro test. Lagged instrumental variables were used in two step panel regressions as described. Models were tidied using broom and broom.mixed [40,41]. Datapoints lying outside 10 standard deviations (sd’s) from the population mean were substituted by temporal kriging (temporal mean substitution) of that states’ data. This was applied to Nebraska data for 2011–2015, lying 11.6 sd’s outside the population mean. Data for multivariable regression was z-transformed to address parameter scale effects. Model reduction from first to final models was by the classical technique of serial omission of the least significant term. Only significant terms are presented. The standard spatial regression model used is the full spatial panel maximum likelihood (spml) model including a maximum likelihood panel looking at main effects over time and using the spatial error structure of Baltagi [42] conducted using the R package splm [39,43,44]. 

The net effect of cannabinoid parameters was summed in multivariable models from matrix multiplication by multiplying the parameter coefficient by the mean value of the covariate and multiplying these measures for interactive terms. The total value for the model was then summed across all covariates in this manner. For robust regression the survey design included the grouping parameter, the inverse probability weighting parameter and the standardized dataset.

### 2.4. Causal Analysis 

Causal analysis was conducted in R using the package ipw to assign inverse probability weights to monthly cannabis use as the exposure of interest [45]. Truncation was not necessary. These weights were then used in weighted mixed effects models performed using the nlme package in R [46]. The strength required of unmeasured confounders to account for the described effects was estimated using E-Values derived from the EValue package in R [47]. *p* < 0.05 was considered significant.

### 2.5. Data Availability Statement

Data including R programming code has been made freely available in the Mendeley Data Archive at URL: http://dx.doi.org/10.17632/tn46tdhc4c.2 (accessed 13 October 2022).

### 2.6. Ethics

The study was approved by the Human Research Ethics Committee of the University of Western Australia on 7 June 2019 No. RA/4/20/4724.

## 3. Results 

### 3.1. Univariate Data

**Figure 1:**
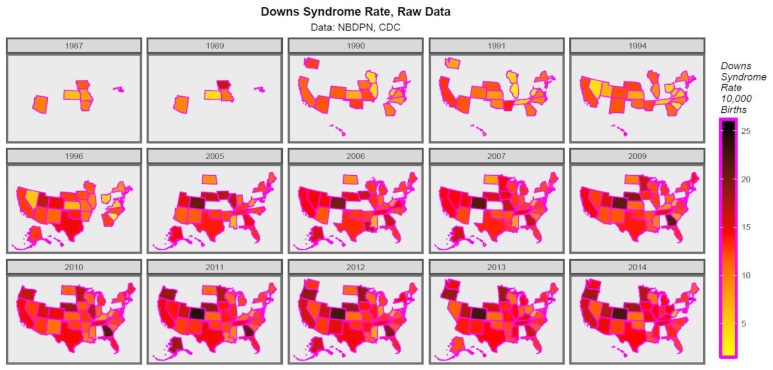
Map-graph of raw Down syndrome rates across USA 1987–2014.

437 data points for the DS rate (DSR) were derived from the published NBDPN database from 1986–1988 to 2012–2016. The mid-year of each annual report was taken as the nominal reference year. This data is displayed in Supplementary Table S1 and map-graphically in Figure 1. In recent years, hotspots are seen to emerge in Colorado and Massachusetts. Supplementary Figure S1 illustrates these changes for estimates of the ETOPFA-corrected DS pregnancy rate calculated as described in Methods. Colorado, Georgia and Massachusetts stand out prominently both before and after correction for ETOPFA’s. The details for the only data source in the world we could identify providing a longitudinal series of ETOPFA rates for DS was the 2015 WARDA report [26]. These data are provided in Supplementary Table S2.

States were divided into quintiles of cannabis use based on the 2015 NSDUH survey as shown in Supplementary Table S3. Colorado, Vermont and Alaska were in Quintile 5 which is the highest cannabis exposure quintile, and Maine, Rhode Island, Oregon and New Hampshire were in the fourth cannabis use quintile.

### 3.2. Bivariate Relationships

**Figure 2:**
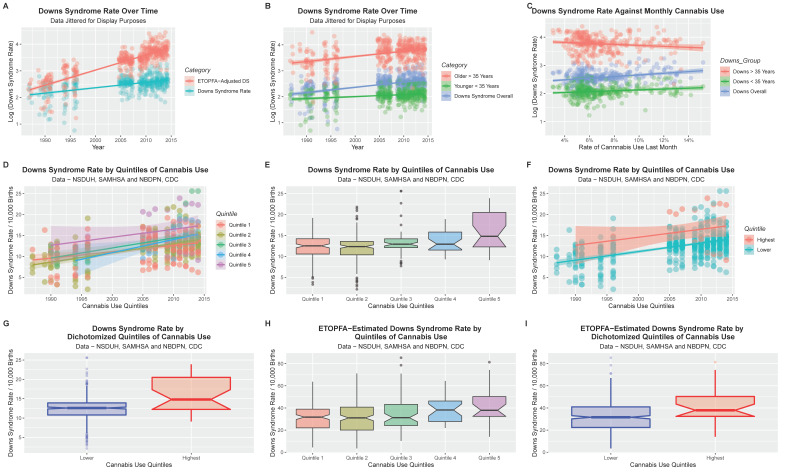
Down Syndrome Univariate analysis. (**A**) DS rate over time for both raw rates and ETOPFA-corrected estimates. (**B**) DS rates over time by maternal age group. (**C**) DS rates as a function of cannabis use. (**D**) DS rates over time by cannabis use quintiles. (**E**) Boxplot of DS rates by cannabis use quintiles. (**F**) DS Rates over time by dichotomized quintiles. Quintiles 1–4 have been collapsed into the “Lower category”. The highest quintile is Quintile 5. (**G**) Boxplot of dichotomized cannabis use quintiles. (**H**) Boxplot of cannabis use quintiles of ETOPFA-corrected DS estimates. (**I**) Boxplot of dichotomized cannabis use quintiles for ETOPFA-corrected DS Rates. Note that non-overlapping notches signifies statistical significance.

Figure 2 shows the DSR categorized in in several ways. Panel A shows a rise over time of both the raw data and the estimates for ETOPFA corrections. Panel B shows a rise in time for the three rates reported by NBDPN, as the overall rate, those born to mothers less than 35 years and those older than this cut-off figure. Time-dependent rises are noted in each case. Panel C plots the DSR against the monthly cannabis use rate and notes rises in the overall and under 35 years groups, but not in the older group. Panel D charts the time course of DSR by each cannabis-use quintile. Importantly the highest cannabis use quintile has DSR’s is obviously higher than the other quintiles with little overlap in the standard error shaded zones. Panel E provides the same information as groups irrespective of time. One reads the chart by noticing where the notches do not overlap for such non-overlapping areas indicate statistical significance. There is therefore an impression of a rise with quintile number. Panel F re-presents the same quintile data showing the highest quintile compared to all of the others, and thus dichotomizes the quintile data. Since the error zones are largely non-overlapping this suggests an important statistically significant difference. These dichotomized quintiles are again presented as boxplots in Panel G where the notches just overlap. Panels H and I present a similar quintile analysis of the ETOPFA-corrected estimates with generally similar findings.

Supplementary Figure S2 presents the DSR as separate panels on a gridded plot. Steeply rising rates in Colorado, Georgia, Illinois, Massachusetts and South Carolina are noted. Supplementary Figure S3 presents these data with each state in its approximate position on the map in a geofacetted display. This display allows the rises in the various states to be grouped by geographical region. This shows a group of states in the midwest which rose sharply—Wisconsin, Illinois, Tennessee and Mississippi, Georgia and Ohio—and a group in the northeast—Massachusetts and Rhode Island. Supplementary Figure S4 makes a similar plot for the ETOPFA-corrected estimates and notes widespread uniform rises across the country which vary only in the extent of the rise.

Supplementary Figure S5 presents the NSUDH data on daily or near daily cannabis use (20–30 days per month) and cannabis use in pregnancy at the national level. Dramatic rises in both indices are noted. As these covariates were not found to be significant in the following regression models they were omitted from final models.

### 3.3. Linear Regressions

Supplementary Table S4 presents linear regression of key covariates of the DSR from these opening data. One notes highly significant terms for time (β-est. = 0.21, (95%C.I. 0.17, 0.25), *p* < 2.2 × 10^−16^), monthly cannabis use (β-est. = 2.97, (1.91, 4.03), *p* = 8.2 × 10^−8^), cannabis use quintiles (β-est. = 3.86, (2.45, 5.27), *p* = 1.2 × 10^−7^), dichotomized cannabis use quintiles (β-est. = 3.54, (2.19, 4.89), *p* = 4.4 × 10^−7^) and time: quintile interactions.

### 3.4. Multiway Panelled Plots

**Figure 3:**
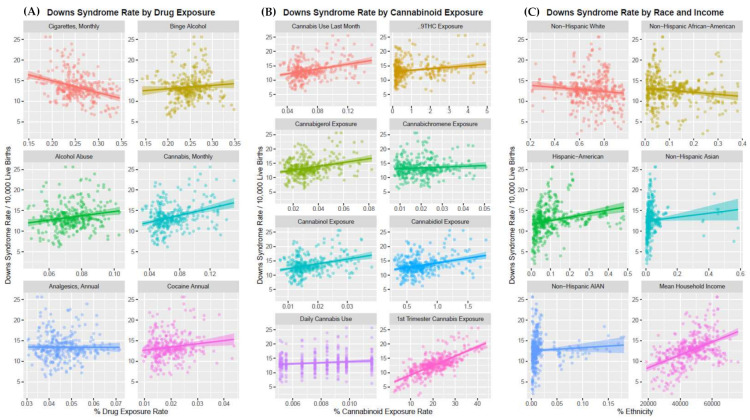
Bivariate plots of the DSR associations. (**A**) Drugs. (**B**) Cannabinoids. (**C**) Ethnicity and Median Household income.

Figure 3 presents plots of the DSR against continuous covariates in each of the four principal domains of interest, (A) drug use, (B) cannabinoid exposure and (C) ethnic background (as defined by the US census profiles for each state) with median household income presented in the final panel of the Panel C. The plots are of interest from several points of view. A strongly positive rising effect is noted with income which is well described in the literature. There appears to be a fall with tobacco use, no relationship with analgesic use, but a rise with alcohol abuse, cocaine and cannabis exposure. Four of the five cannabinoids listed—THC, cannabigerol, cannabichromene, cannabinol-show a positive relationship with DSR as does daily cannabis use and the First Trimester Cannabis Exposure index. A strong ethnic trend amongst Hispanic-Americans is also apparent.

### 3.5. Case Ascertainment

One issue relates to whether the style of case ascertainment of the different birth defects registries may impact the reporting rate of DS. As shown in Supplementary Figure S6 it does appear to, however the direction is the reverse of what would likely be expected with the rate in the active case-finding registries lower than those without. Compared to passive case-finding registries the rate in the active registries is significantly lower (β-est. = −0.10, (−0.17, −0.03), *p* = 0.0080; model F = 4.273, df = 2141, *p* = 0.0158). However, as this variable was not found significant in multivariable models during exploratory modelling it was not considered further. 

### 3.6. Correlograms

Supplementary Figure S7 presents a correlogram for these correlations for drugs and CDC-derived data on maternal age (as the fraction of mothers over 35 years). The correlation coefficient together with its confidence interval appear in the upper triangle and the colour code is from yellow—For strongly positive to maroon strongly negative. The correlogram is sorted along its diagonal by principal component analysis. The correlation cannabis use with DSR is shown as 0.31 (95%C.I. 0.21, 0.40) and the correlation of cannabis use with the ETOPFA-correction for DSR as 0.39 (95%C.I. 0.30, 0.48). The first square in this chart is for “FrOlder35” the fraction of mothers older than 35 years.

Supplementary Figure S8 performs the same role for state-based exposure to cannabinoids. For example, the correlation between state-based THC exposure and ETOPFA-estimates of DSR is 0.51 (95%C.I. 0.42, 0.58).

Supplementary Figure S9 performs a similar role for ethnicity which is weakly correlated, and for ethnic cannabis exposure which for many ethnicities is strongly correlated with both the DSR and ETOPFA-corrected DSR (ETOPFAC-DSR). Hence, for cannabis exposure in the Non-Hispanic African American, Non-Hispanic Caucasian-American, Hispanic-American and Asian-American communities very high correlation coefficients are reported with ETOPFAC-DSR (0.55 (0.47, 0.652). 0.56 (0.48, 0.63), 0.59 (0.52, 0.66), 0.56 (0.48, 0.63) respectively).

### 3.7. Multiple Regression

#### 3.7.1. Panel Regression

Panel regression is a suitable technique to use to regress all of these variables in datasets such as this which have multiple missing data. Supplementary Table S5 presents these results for models lagged at zero, two, three and four years. Main effects were assessed for the five substances, five ethnicities, income and advanced maternal age. Lagged instrumental variables were used for THC exposure and cannabigerol exposure and for the THC exposure of the five ethnicities to account for the mediating effect of these covariates on the main effects. Terms including cannabis are significant from β-est. = 3.38, (1.61, 5.14), *p* = 1.72 × 10^−4^. 

#### 3.7.2. Geospatial Regression

This form of data suggests that geospatial analysis should be applicable to it. However, as no extant geospatial algorithm can cope with missing data it is necessary to impute the missing data by temporal kriging which is an acceptable method. The kriged dataset is shown in Supplementary Table S6 which for the period 2005–2014 lists 322 native points, to which 38 have been added totalling 360 points (with 10.5% kriged). 

**Figure 4:**
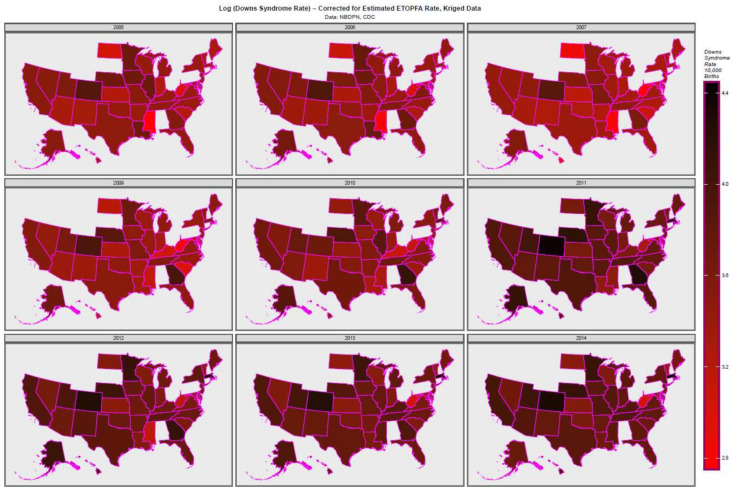
Log of ETOPFA-corrected Down syndrome rates across USA 2005–2014, kriged data.

Supplementary Figure S10 shows this data map-graphically for the raw time series plot, and Figure 4 for the ETOPFA-adjusted estimates. Supplementary Figure S11 shows the geospatial links which were derived from R::spdep and edited as indicated to derive the geospatial weights matrix.

Table 1 shows the results from spatial regression of this data for multivariable models including cannabis, THC and cannabigerol including all the main socioeconomic, ethnicity and drug exposure variables. Table considers first the unadjusted DSR and then the DSR after adjustment for estimated ETOPFA rates. In each case first cannabis, then THC and then cannabigerol are considered. The columns of the right hand of the table list the value of the spatial coefficient rho along with its statistical significance.

12 of the 14 terms for cannabinoids in this Table are positive in direction so on the face of it the effect of cannabinoids appears to be in the positive direction. However, this may be formally determined in multivariable models by the technique of summation of term coefficients by the mean value, multiplying them together for interactive terms, and then adding them across all terms in the model. In this case, since the covariates have all been standardized (by z-transformation) their mean value in each case is zero. Hence, the terms can simply by added directly for all the cannabinoid terms in each model. The sum of these terms is called the Sum of the Cannabinoid Coefficients (SCC) and appears in the right hand column of the table. In all six models this parameter is strongly positive.

### 3.8. Legal Status

**Figure 5:**
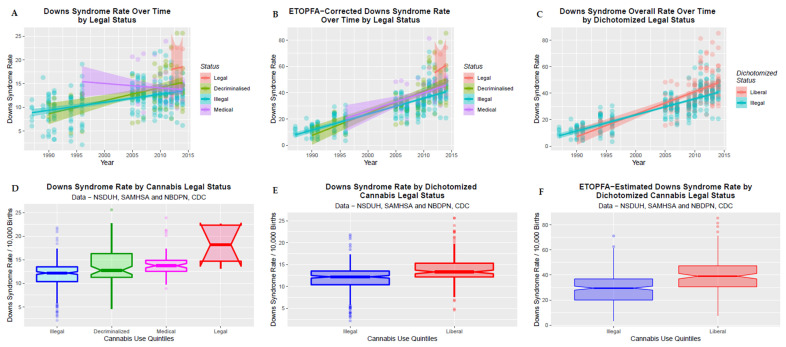
Effect of legal status on DSR. (**A**) DS over time by legal status. (**B**) ETOPFA-corrected estimates of DSR over time by legal status. (**C**) DSR over time by dichotomized legal status. The three regimes representing relaxed cannabis laws are collapsed into the “liberal” category. (**D**) Boxplot of DSR by cannabis legal status. Note that non-overlapping notches signifies statistical significance. (**E**) DSR by dichotomized cannabis legal status. (**F**) ETOPFA-corrected DSR by dichotomized legal status.

It was also of interest to see if the legal status of cannabis might be associated with the DSR. This is illustrated in Figure 5 where the four legal statuses are charted over time in panel A, the ETOPFA-corrected DSR is charted over time in panel B, the dichotomized legal status is charted against the DSR in Panel C, as boxplots in panel D, dichotomized boxplots in panel E and dichotomized boxplots for the ETOPFA-corrected estimates in panel F. In each case apparently highly significant changes are shown.

These changes are quantified in Table 2 which regresses the DSR against each of the parameters shown. Cannabis legalization is shown to be associated with an increased DSR (β-est. = 6.50, (5.02, 8.99), R.R. = 5.97 (3.02, 11.79), *p* = 4.36 × 10^−7^) and when the status is dichotomized by illegal v. more liberal regimes the significance rises (to β-est. = 2.16, (1.50, 2.82), R.R. = 1.81, (1.51, 2.16), *p* = 4.7 × 10^−10^).

### 3.9. Inverse Probability Weighted Mixed Effects Regression

Given the strong evidence established from the geospatial models of a close association across both space and time between DS and survey measurements of drug and cannabinoid exposure the next logical issue related to a formal investigation of whether it may be formally possible to demonstrate a causal relationship. This was facilitated by the use of inverse probability weights in the final kriged model, derived from the ipw Package in R. 

Table 3 presents the results of the fixed effects analysis of inverse probability weighted mixed effects models for three models, a simple additive model, a model interactive in drug exposures and a model interactive in both drug exposures and ethnicities. All covariates in this Table have been standardized by z-transformation. All models include all substance exposures, ethnicities and median household income. For each model covariates are listed in descending order of their coefficient. In addition to the usual mixed effects models parameters the Model Parameter column on the right hand of the table include a Sum of the Cannabinoid Coefficients (SCC) term which has the same meaning as in Table 1. It is noted that in the second and third models the SCC is strongly positive.

Supplementary Table S7 shows the fixed effects of final model outputs from increasingly complex mixed models regressing the DSR against six addictive agents (tobacco, alcohol abuse, opioid analgesics, cocaine, THC and cannabigerol), four races (Caucasian American, African American, Hispanic American and Asian Americans), the ethnic cannabis use index for these four ethnicities and median household income. The first model is an additive model, the second is interactive in terms of the addictive agents (as indicated in Table 3) and the final model has interactions included both amongst the drugs and also amongst the ethnic use of cannabis (as indicated). The best model is the final model (AIC’s of additive and final interactive models 24811.022 vs. 1757.386, ANOVA: df 15 vs. 27, Log Likelihood ratio =747.6357, *p* = 1.50 × 10^−17^). Many highly significant terms including cannabinoids appear in final models for both the substances and ethnic cannabis use indices.

### 3.10. Robust Regression

Robust regression which accounts for model structure has also been employed using the R package survey. In these models the state identity, the dataset and inverse probability weights were all accounted for in the model design term. Table 4 presents these results for the ETOPFA-corrected DSR. In five of the six models terms for cannabis or cannabinoids remain significant in final models.

**Figure 6:**
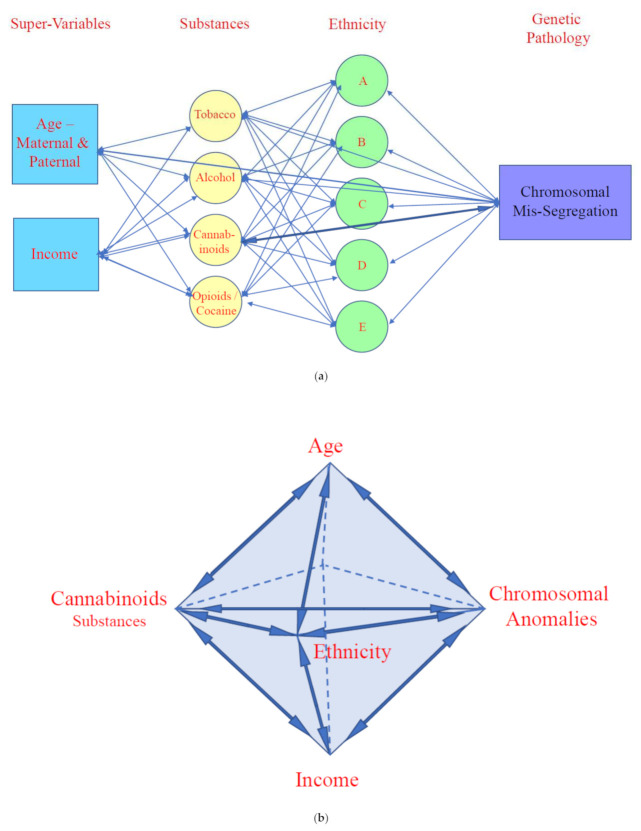
(**a**). Directed Acyclic Graph (DAG) showing presumed causal relationships of covariates in two dimensions (see text for details). (**b**). Three Dimensional Schema representing mutual interaction between four main domains of covariates impacting chromosomal anomalies including direct and indirect effects. Note that each domain impacts the others and the broad and over-arching general effects of age and income supervariables.

Mechanistically these changes may be summarized in the following Figure 6 which presents a Directed Acyclic Graph (DAG) of the various covariates studied. Figure 6a shows four domains namely, Supervariables, Substances, Ethnicity and Genomic pathology. Age is a well recognized super-variable as chromosomal and genetic disorders become more common for many reasons with age. Income is also involved as it affects access to substances and lifestyle factors. All the substances are implicated in genotoxic outcomes, most particularly cannabinoids. A strong link is therefore shown between cannabis and genotoxic outcomes. Ethnicity is also implicated but regression studies show that its effect is largely accounted for by cannabinoid exposure by ethnicity as its effect disappears after adjustment. These various domains are joined by two headed arrows indicating largely two way effects throughout.

However, these effects are not conceptualized in two dimensions. Rather, as shown in Figure 6b each of these four domains is seen as a vertex connected to each of the other vertices as in a double tetrahedron in three dimensions. Hence, all four domains are interconnected both directly and via the other domains. That is both direct (main) effects and interactive and interdependent effects will be seen and described.

### 3.11. Causality and Uncontrolled Confounding

The final question related to quantitation of unmeasured uncontrolled confounding. This was considered using E-Values (from package EValue in R). These are listed in Table 5. One notes that minimum E-Values are generally considered to be potentially causal if they are greater than 1.25 [48]. 52 of the 62 (83.9%) E-Values listed in this table exceed this threshold. For the E-value estimates the median value is 303.98 (IQR 2.50, 2.75 × 10^7^). For the 95% lower bound of the E-value confidence interval the median value is 10.92 (IQR 1.82, 7990). For clarity these E-values are presented in tabular form as a list in Table 6. The nexus between the E-value pairs has been broken in this Table to allow both lists to be presented in consecutive descending order.

### 3.12. Multivariate Regression of Cannabis Legal Status

It was of interest to determine if cannabis legal status was significant after multivariable adjustment. Inverse probability weight linear regression was used for this study. Supplementary Table S8 presents the results of additive and interactive models for the legal status and the dichotomized legal status with the Down syndrome rate as the dependent variable. Table 7 performs the same exercise for the ETOPFA-adjusted DSR in additive and interactive model. These studies showed that in each case legal status continued to be significant in all models after adjustment for the full panel of substance exposure, ethnicity and income covariates.

## 4. Discussion

### 4.1. Main Findings

The study examined socioeconomic, ethnocultural and substance exposure data that may represent key variables underlying the present rise in US DS rate. We found that the rate is rising quite sharply in many US states and particularly so when estimates of ETOPFA-corrected DSR are considered. The DSR also rises with increase of cannabis exposure and cannabis exposure quintile. When considered by formal multivariable regression across both time and space including median household income, state ethnic composition, cannabinoid- and other drug (cigarette, alcohol, analgesic/opioid, cocaine) exposure and the state rate of mothers older than 35 years, terms including tobacco, Δ9THC, cannabigerol and analgesics/opioids were significant in the final model (*p* < 0.001), and terms including ethnicity, income and maternal age were not. 

Relaxation of the laws relating to the cannabis legal status were also related to raised DSR (β-est. = 2.159 ± 0.339, *p* = 4.7 × 10^−10^).

Exploration of the potentially causal nature of the cannabis-DS relationship by the use of inverse probability weighting in mixed effects models and E-Value calculation (which quantitates uncontrolled and unmeasured confounding) showed that the association fulfilled causal criteria with tiny probabilities on weighted mixed effects modelling and significant E-Values in geospatial modelling.

Interestingly our studies linking Down syndrome and cannabis are consistent with prior reports from Hawaii, Colorado, Canada and Australia [6,8,12,13]. The strength of evidence provided in this paper is stronger than that reported previously from other independent datasets and jurisdictions. The present results are reminiscent of recent powerful studies in Europe on this issue [14,15,18].

### 4.2. Pathways and Mechanisms

Various biological pathways have been described which may contribute to the aetiology of chromosomal aneuploidy. In particular cannabis exposure has been shown to interfere with the synthesis and action of tubulin which is the key monomer from which microtubules are polymerized [49]. Microtubules form the cytoskeletal framework upon which chromosomes are segregated during anaphase and the de-railing of chromosomes during their separation can cause micronucleus formation (to which cannabis has long been known to contribute) and chromosomal pulverization [50]. For these reasons cannabis has been described as an indirect genotoxin. Cannabis has been described as deforming human gross sperm morphology [51] and cannabinol and cannabidiol have been linked with sperm chromosomal breaks [52,53,54]. Cannabis has also been described as being highly toxic to primary oocytes with chromosomal breakages, linkages, rings and microsatellites formed after in vitro culture and exposure to Δ9THC [55].

It has also been well established that most DS is related to disturbances of female meiosis I, which is a very prolonged phase which can extend from foetal in utero life to the end of a woman’s reproductive life at age 50 years [56,57,58]. This implies that the female gametes are actually susceptible to genetic damage throughout the woman’s life which implicates on a theoretical basis prenatal, adolescent and young adult cannabinoid exposures as potentially genotoxic and teratogenic.

Cannabis also has quite marked epigenotoxic effects with marked genome-wide alteration of DNA methylation profiles shown in both human and rat sperm [59,60]. This is important as the epigenome is increasingly being understood to effect many genomic features including double-stranded DNA breakage [61,62]. Moreover, epigenomic disruption of key steps in formation of the mitotic spindle, disruption of the post-translational modifications of tubulin which polymerize to form microtubules of the spindle rays, kinetochore function and binding of chromosomes to the microtubules of the mitotic and meiotic spindles, formation of the centromeric chromatin at which the kinetochores will bind, formation of the centrosomes which draw together then ends of the mitotic spindles to form the normal bipolar arrangement which predicts cell division into two daughter cells, have all been now documented in the detailed epigenomic findings of cannabis withdrawal and dependence [63]. These data imply that the marked degree of epigenomic derangement inherent in cannabinoid exposure is likely a key factor driving the chromosomal mis-segregation events underlying reported cannabis-related chromosomal and genetic anomalies in recently population wide studies [6,13,14,17,20,64,65].

Importantly a longitudinal study of the effects of cannabis on the human and rodent epigenome by DNA methylation noted many changes in the machinery of the centromere and kinetochore and damage to the polymerized tubulin polymers of the microtubules of the mitotic spindle induced by cannabis dependence and withdrawal which will greatly increase the error rate of chromosomal segregation, and lead to chromosomal isolation independent of the mitotic spindle, aneuploidy and micronucleus formation [63]. These changes are discussed at length elsewhere [9,15,66,67].

### 4.3. Causal Assignment

Some exposition of the causal inference analysis employed in this paper is appropriate to clarify the techniques employed for the benefit of readers who might be less familiar with the application of modern statistical techniques to causal inference in direct refutation of the “causalophobic” reputation for which classical statistics is so well known [68]. The major short-coming of classical regression techniques in causal assignment has been the potential for unobserved variables to confound the analysis, and in particular the potential for the exposure of interest to be more common amongst the tested group than amongst the control group, a fallacy which results in a comparison of “apples with oranges”. Inverse probability weighting of an observational or ecological population can be applied to the regression model so that the exposure in the two groups becomes equivalent thereby constructing so-called “pseudo-randomized” equally exposed populations, from which causal conclusions can indeed be drawn. This is equally true for continuous variables as was used in the present study. This methodology is further strengthened by the use of robust regression techniques as was employed herein.

Another major way in which causality can be assessed is by the use of “Expected Values”, known as “E-Values” [69]. This is best illustrated by a classical illustration from recent medical history which has been eloquently recounted by one of the foremost causal inferential statisticians of our time Judea Pearl [70]. Prior to the 1950′s lung cancer was a very rare disease. However, with the rise of tobacco and then cigarette smoking lung cancer became common. It was realized before long that cigarette smoking posed a considerable exposure risk with a relative risk elevation amongst smokers of nine-fold. However, the debate was protracted and conflicted since many of the world’s leading scientists—including leading statisticians—smoked tobacco. The debate centred upon whether the observed effect might be confounded by unobserved variables—such as a pre-disposing genetic risk—which might confound the observed relationship. Eventually a genetic risk was indeed identified in 2016 with the identification of a gene which doubled the lung cancer risk. The gridlock was resolved when Robins showed that it was inconceivable that an unobserved confounder variable—which has to be related to both the exposure and the outcome—would be sufficiently powerful to account for all of the observed tobacco effect.

The demonstration in the present study then of many elevated minimum E-Values strongly suggests that the association between cannabis use and Down syndrome is likely to be causal. For example, the present results compares favourably with many modern reports which often have typical E-Values of about 1.25 [48]. However, since both inverse probability weighting and E-values have a number of underlying theoretical assumptions formal demonstration of the causal nature of the link must await further experimental studies. Given the strong evidence presented in the present report such studies should be pursued with high and urgent priority.

Moreover, it is further noted that the present work fulfills all the qualitative Hill criteria of causality as well [71]. Strength of association, consistency amongst studies [6,13,64,65], specificity, temporal sequence (see geotemporospatial analysis), coherence with known data, biological plausibility, dose–response relationships, analogy with similar situations elsewhere [6,13,64,65] and experimental confirmation are all very adequately fulfilled by the epidemiological analyses presented herein and published experimental work as summarized in the Section 4.2 above. These findings may be summarized in the following Table 8.

### 4.4. Implications for Policy

Chromosome 21 is 48 million megabases in length so the now repeated demonstration of strong associations between cannabis use and Down syndrome in a sixth location [6,8,12,13] and in a causal paradigm necessarily implies the implication of cannabis in clinical genotoxic syndromes at the chromosomal megabase scale. The known associations of Down syndrome with congenital heart disease on the one hand and acute lymphoid leukaemia on the other [2,3,4] further imply that adult exposure to cannabinoids is necessarily linked with intergenerational and transgenerational transmission of cannabinoid-related teratogenicity and cannabinoid-related carcinogenicity which are each major aspects of cannabinoid-related genotoxicity. Indeed cannabis has been noted to be an indirect chromosomal clastogen by lab researchers [54,72,73] and has been implicated in a host of genotoxic and epigenotoxic activities as enumerated briefly above [50,52,54,59,72,73,74,75,76,77,78,79,80,81,82,83].

Since data implicate cannabis in heritable genotoxic syndromes it follows that access to cannabinoid products should be restricted and controlled in the same way as other known genotoxic agents including chemotherapeutic cancer drugs and known genotoxins such as thalidomide. Indeed it is of considerable interest that cannabis shares many of the pathophysiological cellular and molecular mechanisms of thalidomide [84,85,86,87,88,89,90] and is also linked with congenital heart defects [91] and limb outgrowth defects [6,13,65,92] just as thalidomide has been [21,84,89,93,94,95,96].

In short the well described implication of cannabis with heritable clinical genotoxic syndromes is a major motivation for carefully controlling the community penetration of cannabinoid products of all types in line with the way known genotoxins are handled, addressed and carefully controlled in every other entry in the pharmacopeia of western medicine. This statement in relation to cannabinoid genotoxicity is strongly supported by the now known causal links relating to cannabinoid neurotoxicity both in the exposed, expressed as high rates of mental illness in young American adults [97], and in their offspring, expressed as high and rapidly increasing rates of child autism across USA [98,99,100].

### 4.5. Time-Dependent Changes in Down Syndrome Rates

It is clear that improved diagnostic techniques are applied to antenatal diagnosis in more recent times so it is possible that there has been a secular rise in the diagnosis of DS which might contribute in part to some of the findings reported. However, the principle results persist even when the uncorrected DSR’s are used (results not shown). Moreover, it may be argued that the rate of termination for anomaly has risen over time. Whilst the present analysis accounts for this it is important also to note that similar—and even more marked-findings were noted in the European dataset where this effect was explicitly included in the raw data themselves [14]. Thus the European data is more robust that the USA data in this respect as the ETOPFA effect could only be estimated for the USA data. Where the ETOPFA effect was known and available the cannabis effect was actually found to be stronger than that documented in the present report [14].

### 4.6. Generalizability

Since the subject of our study was USA, which by many metrics is the world’s leading nation, and is consistent with reports from several other western nations [6,8,12,13,14] and especially from the large European dataset [14], we feel that study findings are generalizable to other developed nations. However, in view of the rapidly expanding nature of cannabis commercialization, and the widespread misunderstanding of its supposedly benign nature, more research needs to be performed at both the genotoxicity / epigenotoxicity level, and at the epidemiological level. NBDPN-CDC recently published an analysis of gastroschisis at the US county level [101] and a similar high definition geospatiotemporal investigation in the area of cannabis and DS is urgently required. This highly generalizable view is supported by similar reports from multiple other jurisdictions including Hawaii, Colorado, Australia, Canada and most recently Europe [6,13,14,17,20,64,65].

### 4.7. Strengths and Limitations

This study has various strengths and limitations. Its strengths relate to its study of the USA providing the best public data in the world, its population-census wide nature of both its underlying population and birth defect data, its use of the NSDUH which is a robust widely used nationally representative sample, the use of a wide spectrum of covariates, and the application of geospatial and causal inference statistical techniques to this area for the first time to our knowledge. Its limitations relate mainly to its design and the unavailability of individual case-level data. This implies that strictly causal relationships cannot be established. Nevertheless, the present ecological report does pursue this line of investigation as far as this is possible with population level data. Moreover, cannabis use may be associated with other covariates of teratological significance such as tobacco and alcohol use. While these have been adjusted for herein using regression techniques some residual confounding might still exist. Inverse probability weighting minimized this effect. The use of E-Values quantifies the extent to which such effects may be significant. As demonstrated uncontrolled confounding does not perturb the major conclusions emerging from geospatial models. Cannabis use has been shown in many studies to be inversely associated with maternal age so consideration of this variable would likely increase the observed effects. For these reasons further research in this area is urgently required.

### 4.8. Conclusions

Our interpretation of study findings is that there exist solid grounds for accepting a positive epidemiological association between cannabis exposure and DS across both space and time in the present study and also in many other recent series. These results from USA are consistent with similar data from several other states and nations [6,13,14,17,20,64,65] and are further strengthened in the context of the known genotoxicity and epigenotoxicity of multiple ethnobiological cannabinoids. Clearly more research in this area is necessary. Furthermore, in view of its public health implications a greatly augmented public education campaign of current empirical findings should be encouraged following the public health model and approach to tobacco.

## Figures and Tables

**Table 1:** Geospatial spreml Regression of Down Syndrome Rate on Drugs, Cannabinoids, Race and Income.

Parameter			Model Spatial Parameter, Rho		SCC
Parameter	Estimate (C.I.)	*p*-Value	Value	*p*-Value	
					
***Down Syndrome Rate***					
					
***Cannabis***					
*spml* (DS_Rate~Cigarettes * Cannabis_Exposure * Alcoholism + Analgesics + Cocaine + MHY + 5_Races + AdvMatlAge)					
Asian	0.48 (0.10, 0.86)	0.0140	−0.2453	0.0003	0.5578
Hispanic	0.44 (0.20, 0.67)	2.86 × 10^−4^			
NHWhite	0.39 (0.13, 0.65)	2.86 × 10^−3^			
Cannabis	0.36 (0.20, 0.52)	9.01 × 10^−6^			
NHBlack	0.33 (0.12, 0.54)	0.0022			
Cannabis: Alcoholism	0.20 (0.10, 0.30)	0.0001			
Median Household Income	0.17 (0.02, 0.32)	0.0290			
Alcoholism	0.15 (0.07, 0.24)	7.30 × 10^−4^			
Cigarettes: Alcoholism	0.08 (0.01, 0.15)	0.0314			
Cigarettes	−0.16 (−0.29, −0.03)	0.0203			
Analgesics	−0.18 (−0.30, −0.06)	0.0045			
Mothers_Older_35_Years	−0.54 (−0.77, −0.31)	4.92 × 10^−6^			
					
***THC***					
*spml* (DS_Rate~Cigarettes * THC_Exposure * Alcoholism + Analgesics + Cocaine + MHY + 5_Races + AdvMatlAge)					
THC_Exposure	0.32 (0.16, 0.47)	5.51 × 10^−5^	-0.2759	4.78 × 10^−5^	0.2588
Median Household Income	0.20 (0.07, 0.33)	0.0021			
Hispanic	0.18 (0.08, 0.27)	0.0002			
THC_Exposure: Alcoholism	0.11 (0.00, 0.21)	0.0496			
Alcoholism	0.09 (0.00, 0.18)	0.0394			
Analgesics	−0.16 (−0.27, −0.05)	0.0047			
Cigarettes: THC_Exposure: Alcoholism	−0.16 (−0.25, −0.08)	0.0001			
Mothers_Older_35_Years	−0.26 (−0.40, −0.12)	0.0004			
					
					
***Cannabigerol***					
*spml* (DS_Rate~Cigarettes * CBG_Exposure * Alcoholism + Analgesics + Cocaine + MHY + 5_Races + AdvMatlAge)					
Hispanic	0.42 (0.18, 0.65)	0.0005	−1.6944	1.25 × 10^−5^	0.4159
CBG_Exposure	0.41 (0.23, 0.59)	5.15 × 10^−6^			
Asian	0.41 (0.03, 0.78)	0.0325			
NHWhite	0.34 (0.09, 0.60)	0.0084			
NHBlack	0.31 (0.10, 0.52)	0.0035			
Median Household Income	0.19 (0.04, 0.34)	0.0153			
Alcoholism	0.14 (0.05, 0.23)	0.0024			
CBG_Exposure: Alcoholism	0.12 (0.01, 0.22)	0.0379			
Cigarettes: CBG_Exposure: Alcoholism	−0.11 (−0.20, −0.02)	0.0148			
Cigarettes	−0.14 (−0.28, 0.00)	0.0439			
Analgesics	−0.16 (−0.28, −0.04)	0.0086			
Mothers_Older_35_Years	−0.51 (−0.74, −0.29)	9.26 × 10^−6^			
					
					
***Estimated Corrected Down Syndrome Rate***					
					
***Cannabis***					
*spml(DS_Rate~* Cigarettes * Cannabis_Exposure * Alcoholism + Analgesics + Cocaine + MHY + 5_Races + AdvMatlAge)					
Asian	0.35 (0.05, 0.64)	0.0221	-0.251266	0.0002	0.4128
Hispanic	0.28 (0.10, 0.46)	0.0026			
NHWhite	0.27 (0.06, 0.47)	0.0098			
Cannabis	0.25 (0.13, 0.37)	5.22 × 10^−5^			
NHBlack	0.21 (0.05, 0.37)	0.0111			
Cannabis: Alcoholism	0.16 (0.09, 0.24)	4.64 × 10^−5^			
Alcoholism	0.14 (0.07, 0.21)	8.48 × 10^−5^			
Cigarettes: Alcoholism	0.07 (0.02, 0.13)	0.0108			
Analgesics	-0.13 (-0.22, -0.03)	0.0087			
Cigarettes	-0.16 (-0.26, -0.06)	0.0025			
Mothers_Older_35_Years	-0.33 (-0.50, -0.17)	9.69 × 10^−5^			
					
					
***THC***					
*spml(DS_Rate~* Cigarettes * THC_Exposure * Alcoholism + Analgesics + Cocaine + MHY + 5_Races + AdvMatlAge)					
**THC_Exposure**	0.30 (0.16, 0.43)	2.72 × 10^−5^	−0.2798	3.67 × 10^−5^	0.4344
Asian	0.29 (0.00, 0.58)	0.0500			
Hispanic	0.25 (0.07, 0.43)	0.0055			
NHWhite	0.23 (0.04, 0.43)	0.0200			
NHBlack	0.18 (0.03, 0.34)	0.0224			
THC_Exposure: Alcoholism	0.14 (0.06, 0.22)	0.0006			
Alcoholism	0.13 (0.06, 0.20)	0.0002			
Cigarettes: Alcoholism	0.08 (0.02, 0.13)	0.0101			
Analgesics	−0.11 (−0.20, −0.02)	0.0175			
Cigarettes	−0.15 (−0.25, −0.05)	0.0026			
Mothers_Older_35_Years	−0.30 (−0.46, −0.15)	0.0002			
					
					
***Cannabigerol***					
spml(DS_Rate~Cigarettes * CBG_ Exposure * Alcoholism + Analgesics + Cocaine + MHY + 5_Races + AdvMatlAge)					
CBG_Exposure	0.22 (0.12, 0.33)	1.24 × 10^−5^	−0.067	0.3253	0.3114
MHY	0.11 (0.01, 0.22)	0.0408			
CBG_Exposure: Alcoholism	0.09 (0.01, 0.17)	0.0281			
Mothers_Older_35_Years	−0.18 (−0.29, −0.06)	0.0029			
Cigarettes	−0.19 (−0.27, −0.11)	5.92 × 10^−6^			

Abbreviations: 5_Races:—Caucasian American + African American + Hispanic American + Asian American + American Indian/Alaskan, Native, rho:-Spatial autoregressive parameter, SCC—Sum Cannabinoid Coefficients.

**Table 2:** Legal Status—Bivariate Linear Regressions.

Parameter	Parameters		Model Parameters			
	Estimate (C.I.)	*p*-Value	R-Squared	F	dF	P
						
***lm(Downs_Rate~Legal_Status)***						
Status-Legal	6.5 (4.02, 8.99)	4.36 × 10^−7^	0.1085	18.48	3428	2.72 × 10^−11^
Status-Decriminalised	1.70 (0.89, 2.52)	5.04 × 10^−5^				
Status-Medical	2.27 (1.36, 3.19)	1.40 × 10^−6^				
						
***lm(Downs_Rate~Dichotomized_Legal_Status)***						
Liberal_Status	2.16 (1.50, 2.82)	4.68 × 10^−10^	0.0843	40.66	1430	4.68 × 10^−10^
						
lm(Downs_Rate~Year * Legal_Status)						
Year	0.17 (0.13, 0.21)	5.2 × 10^−14^	0.2607	22.71	7424	<2.2 × 10^−16^
Status-Medical	529.63 (48.63, 1010.62)	0.0315				
Year:Status-Medical	−0.26 (−0.5, −0.02)	0.0319				
Year:Status-Decriminalised	0.10 (0.00, 0.21)	0.0569				
Status-Decriminalised	−203.45 (−413.39, 6.49)	0.0582				
						
lm(Downs_Rate~Year * Dichotomized_Legal_Status)						
Liberal_Status	0 (0, 0)	8.2 × 10^−5^	0.2359	67.54	2429	<2.2 × 10^−16^

**Table 3:** Mixed Effects Model with Inverse Probability Weights—Fixed Effects. Standardized Covariates.

Parameter			Model	
Covariate	Estimate (C.I.)	*p*-Value	Model Parameter	Value
				
***Additive Model***				
*lme(Downs_Rate~Cigarettes + Alcohol.Abuse + THC.Exposure +* *Cannabigerol* *.Exposure + Analgesics + Cocaine + 4_Races+ Median.HH.Income, random = ~1|id, weights = ~sw)*				
Hispanic	33.2 (32, 34.4)	5.66 × 10^−159^	AIC	393.005
THC.Exposure	4.28 (4.19, 4.37)	9.29 × 10^−226^	B IC	3983.781
Cocaine	0.99 (0.95, 1.02)	2.31 × 10^−164^	LogLik	−1954.003
Alcoholism	0.51 (0.43, 0.59)	1.88 × 10^−31^	S.D.	51446.470
Analgesics	−0.16 (−0.19, −0.13)	1.92 × 10^−21^	SCC	−0.020
Cigarettes	−0.36 (−0.4, −0.32)	4.24 × 10^−43^		
Median Household Income	−1 (−1.04, −0.96)	4.58 × 10^−153^		
CBG.Exposure	−4.3 (−4.42, −4.17)	2.91 × 10^−186^		
NHWhite	−8.24 (−8.78, −7.7)	2.01 × 10^−92^		
Asian	−21.6 (−22.4, −20.8)	6.01 × 10^−159^		
				
***Drug Interactive Model***				
lme(Downs_Rate~Cigarettes*Alcohol.Abuse*THC.Exposure* *Cannabigerol* *.Exposure* *+* *Analgesics + Cocaine + 4_Races+ Median.HH.Income, random = ~1|id, weights = ~sw)*				
Alcoholism: THC.Exposure	4.98 (4.2, 5.76)	3.31 × 10^−29^	AIC	3252.770
Cigarettes: Alcoholism: THC.Exposure	3.12 (2.51, 3.73)	1.41 × 10^−20^	B IC	3336.416
THC.Exposure	2.72 (2.47, 2.96)	1.21 × 10^−64^	LogLik	-1604.385
Cigarettes: THC.Exposure	1.81 (1.56, 2.05)	8.80 × 10^−36^	S.D.	23106.340
Hispanic	1.14 (0.69, 1.6)	1.11 × 10^−6^	SCC	**2.196**
NHWhite	0.85 (0.62, 1.07)	1.29 × 10^−12^		
Asian	0.83 (0.26, 1.41)	4.47 × 10^−3^		
Cigarettes	0.29 (0.18, 0.41)	9.33 × 10^−7^		
Analgesics	0.18 (0.11, 0.24)	2.01 × 10^−7^		
Median Household Income	−0.24 (−0.34, −0.14)	2.15 × 10^−6^		
Alcoholism: THC.Exposure: CBG.Exposure	−0.4 (−0.68, −0.12)	4.59 × 10^−3^		
Cigarettes: Alcoholism: THC.Exposure: CBG.Exposure	−0.45 (−0.75, −0.15)	3.68 × 10^−3^		
Alcoholism	−0.57 (−0.71, −0.44)	5.89 × 10^−16^		
Cigarettes: CBG.Exposure	−0.66 (−1.04, −0.28)	7.76 × 10^−4^		
Cigarettes: THC.Exposure: CBG.Exposure	−0.77 (−0.87, −0.67)	3.38 × 10^−37^		
THC.Exposure: CBG.Exposure	−0.77 (−0.94, −0.61)	6.06 × 10^−18^		
CBG.Exposure	−1.32 (−1.72, −0.91)	6.68 × 10^−10^		
Cigarettes: Alcoholism: CBG.Exposure	−2.13 (−2.74, −1.52)	3.82 × 10^−11^		
Alcoholism: CBG.Exposure	−3.94 (−4.71, −3.17)	1.55 × 10^−20^		
				
				
***Drug and Ethnic Interactive Model***				
lme(Downs_Rate~Cigarettes*Alcohol.Abuse*THC.Exposure*Cannabigerol.Exposure *+* *Analgesics + Cocaine +* NHWhite*NHBlack*Asian*Hispanic+ *Median.HH.Income, random = ~1|id, weights = ~sw)*				
NHBlack: Hispanic: Asian	11.4 (8.43, 14.4)	9.42 × 10^−13^	AIC	3198.600
NHWhite: NHBlack: Asian	8.29 (5.69, 10.9)	1.29 × 10^−9^	B IC	3311.930
NHWhite: NHBlack: Hispanic: Asian	6.43 (4.6, 8.25)	2.82 × 10^−11^	LogLik	-1569.300
NHBlack: Asian	5.01 (1.89, 8.14)	1.75 × 10^−3^	S.D.	17081.080
NHWhite: Asian	4.31 (2.55, 6.07)	2.27 × 10^−6^	SCC	**4.749**
Alcoholism: THC.Exposure	4.09 (3.38, 4.8)	4.77 × 10^−25^		
NHBlack: Hispanic	3.75 (1.06, 6.45)	6.57 × 10^−3^		
Cigarettes: Alcoholism: THC.Exposure	3.16 (2.36, 3.95)	1.07 × 10^−13^		
THC.Exposure	2.66 (2.13, 3.19)	4.61 × 10^−20^		
Cigarettes: THC.Exposure	2.46 (1.91, 3.01)	1.10 × 10^−16^		
Cigarettes	0.35 (0.21, 0.49)	1.00 × 10^−6^		
Analgesics	0.13 (0.06, 0.2)	2.03 × 10^−4^		
Median Household Income	−0.23 (−0.34, −0.12)	4.78 × 10^−5^		
Cocaine	−0.3 (−0.46, −0.14)	2.92 × 10^−4^		
Alcoholism	−0.67 (−0.79, −0.54)	1.89 × 10^−21^		
CBG.Exposure	−0.67 (−1.21, −0.13)	1.46 × 10^−^^2^		
Cigarettes: CBG.Exposure	−1.01 (−1.56, −0.45)	4.04 × 10^−4^		
Alcoholism: THC.Exposure: CBG.Exposure	−1.1 (−1.42, −0.79)	2.54 × 10^−11^		
Cigarettes: Alcoholism: THC.Exposure: CBG.Exposure	−1.19 (−1.52, −0.85)	2.01 × 10^−11^		
Cigarettes: THC.Exposure: CBG.Exposure	−1.4 (−1.63, −1.17)	3.05 × 10^−26^		
THC.Exposure: CBG.Exposure	−1.69 (−2.02, −1.36)	1.15 × 10^−20^		
Cigarettes: Alcoholism: CBG.Exposure	−1.74 (−2.45, −1.03)	2.23 × 10^−6^		
Asian	−1.96 (−3.67, −0.26)	2.40 × 10^−2^		
Alcoholism: CBG.Exposure	−2.57 (−3.26, −1.88)	2.38 × 10^−12^		
NHWhite: NHBlack	−2.65 (−4.32, −0.98)	1.94 × 10^−3^		
Hispanic: Asian	−5.36 (−7.65, −3.07)	6.25 × 10^−6^		
NHWhite: Hispanic	−5.68 (−6.72, −4.64)	7.73 × 10^−23^		

Abbreviations: AIC—Akiake Information Criterion, BIC—Bayesian Information Criterion, LogLik—Log likelihood at model optimization, S.D.—Model Standard Deviation, S.C.C.—Sum of Cannabinoid Coefficients.

**Table 4:** Robust Regression Results (from R Survey Package).

Parameter			Model	
Term	Est. (C.I.)	*p* Value	Parameter	Value
				
**Drugs Additive**				
*svyglm(Downs.ETOPFA.Est.Rate~ Cigarettes + Alcoholism + THC.Exposure + CBG.Exposure + Analgesics + Cocaine)*				
Analgesics	1.19 (0.75, 1.64)	5.13 × 10^−7^	AIC	−101,077
Cocaine	−1.11 (−1.25, −0.97)	4.03 × 10^−30^	BIC	16.3
			Deviance	33.2
				
**Ethnic THC Exposure**				
svyglm(Downs.ETOPFA.Est.Rate~NHWhite.THC.Exposure + NHBlack.THC.Exposure * Hispanic.THC.Exposure * Asian.THC.Exposure)				
Hispanic.THC.Exposure	0.68 (0.58, 0.78)	8.08 × 10^−32^	AIC	2225
			BIC	61
			Deviance	2.06
				
**Cannabis-Full Additive Model**				
*svyglm(Downs.ETOPFA.Est.Rate~Cigarettes + Alcoholism + Cannabis.Exposure + Analgesics + Cocaine +*				
*NHWhite + NHBlack + Hispanic + NHAsian + Median.HH.Income)*				
Cannabis.Exposure	0.41 (0.18, 0.64)	5.33 × 10^−04^	AIC	2301
Hispanic	0.39 (0.07, 0.71)	0.0156	BIC	88.3
NHWhite	0.36 (0.06, 0.67)	0.0201	Deviance	49.3
Median.HH.Income	0.31 (0.07, 0.55)	0.0124		
NHBlack	0.3 (0.05, 0.55)	0.0201		
Cocaine	−0.53 (−0.78, −0.28)	3.50 × 10^−5^		
				
**Cannabinoids-Full Additive Model**				
*svyglm(Downs.ETOPFA.Est.Rate~Cigarettes + Alcoholism + THC.Exposure + CBG.Exposure + Analgesics + Cocaine +*				
*NHWhite + NHBlack + Hispanic + NHAsian + Median.HH.Income)*				
THC.Exposure	0.33 (0.14, 0.52)	0.0006	AIC	2279
Median.HH.Income	0.22 (0.01, 0.42)	0.0420	BIC	72.8
Cocaine	−0.34 (−0.65, −0.03)	0.0293	Deviance	47.3
				
**Cannabinoids-Full Model-Interactive Substances**				
svyglm(Downs.ETOPFA.Est.Rate~Cigarettes * Cannabis.Exposure * Alcoholism + Analgesics + Cocaine +				
*NHWhite + NHBlack + Hispanic + NHAsian + Median.HH.Income)*				
Cigarettes:Cannabis.Exposure	0.53 (0.45, 0.61)	4.60 × 10^−31^	AIC	2257
Cigarettes	0.16 (0.01, 0.32)	3.88 × 10^−02^	BIC	71.0
			Deviance	49.4
				
**Cannabinoids-Full Model-Substances & Ethnicity**				
svyglm(Downs.ETOPFA.Est.Rate~Cigarettes * Cannabis.Exposure * Alcoholism + Analgesics + Cocaine +				
NHWhite + NHBlack * Hispanic * NHAsian + Median.HH.Income)				
NHBlack:Hispanic:NHAsian	0.81 (0.19, 1.44)	0.0112	AIC	2003
NHBlack	0.58 (0.28, 0.89)	0.0002	BIC	104
NHBlack:Hispanic	0.57 (0.11, 1.03)	0.0161	Deviance	53.4
Alcoholism	0.53 (0.24, 0.82)	0.0003		
Median.HH.Income	0.47 (0.19, 0.75)	0.0012		
Hispanic	0.39 (0.13, 0.66)	0.0040		
Cigarettes	0.28 (0.09, 0.48)	0.0038		
Analgesics	0.22 (0.02, 0.43)	0.0354		
Cigarettes:Cannabis.Exposure	0.21 (0.03, 0.4)	0.0233		
Cigarettes:Alcoholism	−0.36 (−0.64, −0.08)	0.0124		
Cocaine	−0.45 (−0.75, −0.16)	0.0029		

**Table 5:** Selected E-Values.

Parameter	Table	Regression Coefficient (C.I.)	R.R. (C.I.)	eValues
				
***Linear Regression***	eTable 4			
Downs over Time	eTable 4	0.21 (0.17, 0.25)	1.067 (1.055, 1.079)	1.33, 1.29
Downs by Monthly Cannabis Use	eTable 4	2.97 (1.91, 4.03)	2.38 (1.72, 3.31)	4.21, 2.83
Downs by Cannabis Use Quintile	eTable 4	3.86 (2.45, 5.27)	2.31 (1.59, 3.37)	4.05, 2.56
Downs by Cannabis Use Quintile Dichotomized	eTable 4	3.54 (2.19, 4.89)	2.07 (1.44, 2.97)	3.56, 2.25
				
***Legal Relationship***	eTable 8			
Legal v Illegal Status	eTable 8	6.50 (4.02, 8.99)	5.97 (3.02, 11.79)	11.41, 5.49
Medical v Illegal Status	eTable 8	2.27 (1.36, 3.19)	1.86 (1.45, 2.39)	3.14, 2.27
Decriminalized v Illegal Status	eTable 8	1.70 (0.89, 2.52)	1.61 (1.29, 2.02)	2.60, 1.90
Time * Decriminalized Status	eTable 8	529.63 (48.63, 1010.62)	Inf (5.6 × 10^70^, Inf)	Inf, 1.1 × 10^71^
Liberal v Illegal Status (Dichotomized)	eTable 8	2.16 (1.50, 2.82)	1.81 (1.51, 2.16)	3.01, 2.39
				
***Geotemporospatial Regression***	Table 1			
Cannabis	Table 1	0.36 (0.20, 0.52)	1.67 (1.33, 2.10)	2.79, 2.00
Cannabis: Alcoholism	Table 1	0.20 (0.10, 0.30)	1.32 (1.15, 1.54)	1.99, 1.56
THC_Exposure	Table 1	0.32 (0.16, 0.47)	1.56 (1.26, 1.94)	2.50, 1.82
THC_Exposure: Alcoholism	Table 1	0.11 (0.00, 0.21)	1.16 (1.00, 1.35)	1.60, 1.02
CBG_Exposure	Table 1	0.41 (0.23, 0.59)	1.78 (1.39, 2.31)	2.99, 2.14
CBG_Exposure: Alcoholism	Table 1	0.12 (0.01, 0.22)	1.17 (1.01, 1.38)	1.64, 1.11
Cannabis	Table 1	0.25 (0.13, 0.37)	1.57 (1.26, 1.97)	2.53, 1.84
Cannabis: Alcoholism	Table 1	0.16 (0.09, 0.24)	1.35 (1.16, 1.56)	1.04, 1.61
THC _ Exposure	Table 1	0.30 (0.16, 0.43)	1.71 (1.33, 2.20)	2.81, 1.99
THC _ Exposure: Alcoholism	Table 1	0.14 (0.06, 0.22)	1.29 (1.11, 1.49)	1.90, 1.47
CBG_Exposure	Table 1	0.22 (0.12, 0.33)	1.67 (1.31, 2.13)	2.73, 1.96
CBG_Exposure: Alcoholism	Table 1	0.09 (0.01, 0.17)	1.16 (1.02, 1.33)	1.60, 1.14
				
***Mixed Effects iptw Regression***				
THC.Exposure	Table 2	4.28 (4.19, 4.37)	1.00007 (1.00007, 1.00007)	1.0087, 1.0086
Alcoholism: THC.Exposure	Table 2	4.98 (4.2, 5.76)	1.0019 (1.00017, 1.00022)	1.014, 1.013
Cigarettes: Alcoholism: THC.Exposure	Table 2	3.12 (2.51, 3.73)	1.0001 (1.00009, 1.0001)	1.011, 1.01
THC.Exposure	Table 2	2.72 (2.47, 2.96)	1.0001 (1.00009, 1.0001)	1.01, 1.009
Cigarettes: THC.Exposure	Table 2	1.81 (1.56, 2.05)	1.00007 (1.00006, 1.00008)	1.008, 1.0079
Alcoholism: THC.Exposure	Table 2	4.09 (3.38, 4.8)	1.002 (1.0001, 1.0002)	1.014, 1.013
Cigarettes: Alcoholism: THC.Exposure	Table 2	3.16 (2.36, 3.95)	1.00016 (1.00013, 1.00021)	1.013, 1.011
THC.Exposure	Table 2	2.66 (2.13, 3.19)	1.0001 (1.0001, 1.0002)	1.012, 1.010
Cigarettes: THC.Exposure	Table 2	2.46 (1.91, 3.01)	1.0001 (1.0001, 1.0001)	1.011, 1.010
				
***Linear Modelling of Multivariate Legal Status***				
*Downs Rate*				
THC.Exposure	Table S8	0.37 (0.08, 0.664)	1.28 × 10^3^ (4.88, 3.33 × 10^5^)	2.55 × 10^3^, 9.24
Status-Decriminalised	Table S8	0.39 (0.176, 0.611)	1.95 × 10^3^ (31.13, 1.22 × 10^5^)	3.89 × 10^3^, 61.76
Status-Medical	Table S8	0.38 (0.0659, 0.697)	1.54 × 10^3^ (3.77, 6.35 × 10^5^)	3.09 × 10^3^, 7.00
CBG.Exposure	Table S8	0.65 (0.414, 0.886)	7.44 × 10^8^ (4.66 × 10^5^, 1.19 × 10^12^)	1.49 × 10^9^, 9.31 × 10^5^
Status-Decriminalised	Table S8	2.47 (2.13, 2.81)	5.40 × 10^5^ (4.00 × 10^3^, 7.29 × 10^7^)	1.08 × 10^6^, 7.99 × 10^3^
Status-Medical	Table S8	0.42 (0.263, 0.577)	5.16 × 10^33^ (1.32 × 10^29^, 2.02 × 10^38^)	1.03 × 10^34^, 2.64 × 10^29^
Cigarettes: CBG.Exposure	Table S8	0.52 (0.052, 0.994)	1.37 × 10^7^ (5.72, 3.31 × 10^13^)	2.75 × 10^7^, 10.92
Cigarettes: THC.Exposure: CBG.Exposure	Table S8	0.39 (0.165, 0.609)	1.91 × 10^5^ (184.19, 1.99 × 10^8^)	3.82 × 10^5^, 367.89
THC.Exposure	Table S8	0.38 (0.0965, 0.653)	1.38 × 10^3^ (6.76, 2.81 × 10^5^)	2.75 × 10^3^, 13.02
Dichotomous.Status-Liberal	Table S8	0.39 (0.212, 0.567)	1.84 × 10^3^ (61.40, 5.53 × 10^4^)	3.68 × 10^3^, 122.30
CBG.Exposure	Table S8	0.96 (0.703, 1.21)	3.46 × 10^11^ (3.26 × 10^8^, 3.67 × 10^14^)	6.93 × 10^11^, 6.54 × 10^8^
Dichotomous.Status-Liberal	Table S8	0.82 (0.663, 0.974)	7.87 × 10^9^ (1.06 × 10^8^, 5.80 × 10^11^)	1.57 × 10^10^, 2.13 × 10^8^
Cigarettes: Alcoholism: THC.Exposure	Table S8	1.13 (0.864, 1.39)	4.51 × 10^13^ (3.07 × 10^10^, 6.63 × 10^16^)	9.02 × 10^13^, 6.14 × 10^10^
				
*ETOPFA-Est. Downs Rate*				
Status-Decriminalised	Table 6	0.28 (0.113, 0.44)	1.17 × 10^3^ (18.35, 7.48 × 10^3^)	2.34 × 10^3^, 36.19
Cannabis.Exposure	Table 6	0.2 (0.0917, 0.301)	152.24 (10.65, 2.17 × 10^3^)	303.98, 20.79
Status-Decriminalised	Table 6	0.27 (0.107, 0.435)	977.51 (15.41, 6.19 × 10^4^)	1.95 × 10^3^, 30.32
THC.Exposure	Table 6	1.65 (1.42, 1.88)	267.50 (18.46, 3.87 × 10^3^)	534.51, 36.41
Cigarettes: Alcoholism: THC.Exposure	Table 6	0.66 (0.546, 0.772)	3.62 × 10^13^ (1.75 × 10^11^, 7.47 × 10^15^)	7.25 × 10^13^, 3.51 × 10^11^
Cigarettes: Alcoholism: THC.Exposure: CBG.Exposure	Table 6	0.63 (0.458, 0.8)	8.75 × 10^12^ (2.81 × 10^9^, 2.72 × 10^16^)	1.75E+13, 5.62 × 10^9^
CBG.Exposure	Table 6	0.59 (0.424, 0.759)	1.44 × 10^12^ (5.38 × 10^8^, 3.88 × 10^15^)	2.89 × 10^12^, 1.07 × 10^9^
Cigarettes: CBG.Exposure	Table 6	0.55 (0.151, 0.943)	1.79 × 10^11^ (1.46 × 10^3^, 2.21 × 10^19^)	3.59 × 10^11^, 2.95 × 10^3^
Cigarettes: THC.Exposure: CBG.Exposure	Table 6	0.33 (0.0888, 0.568)	5.60 × 10^6^ (68.97, 4.56 × 10^11^)	1.12 × 10^7^, 137.45
Status-Medical	Table 6	0.23 (0.121, 0.34)	8.90 × 10^33^ (2.09 × 10^29^, 3.78 × 10^38^)	1.78 × 10^34^, 4.19 × 10^29^
Status-Decriminalised	Table 6	0.23 (0.121, 0.34)	5.66 × 10^4^ (327.53, 9.78 × 10^6^)	1.13 × 10^5^, 654.55
Dichotomous Status-Liberal	Table 6	0.39 (0.212, 0.567)	165.93 (6.01, 4.58 × 10^3^)	331.35, 11.50
Cannabis.Exposure	Table 6	0.38 (0.0965, 0.653)	75.43 (5.78, 967.81)	150.36, 11.23
THC.Exposure	Table 6	0.44 (0.237, 0.65)	1.04 × 10^5^ (497.78, 2.18 × 10^7^)	2.08 × 10^5^, 995.06
Dichotomous Status-Liberal	Table 6	0.26 (0.124, 0.387)	782.59 (26.10, 2.34 × 10^4^)	1.56 × 10^3^, 51.69
Cigarettes: Alcoholism: THC.Exposure: CBG.Exposure	Table 6	1.22 (0.999, 1.43)	3.60 × 10^20^ (8.53 × 10^16^, 1.52x 10^24^)	7.230 × 10^20^, 1.71E+17
Cigarettes: Alcoholism: THC.Exposure	Table 6	1.18 (0.779, 1.59)	7.63 × 10^19^ (1.213 × 10^13^, 4.69 × 10^26^)	1.53 × 10^2^, 2.48 × 10^13^
THC.Exposure	Table 6	1 (0.736, 1.27)	7.07 × 10^16^ (2.51 × 10 × 10^12^, 1.99 × 10^21^)	1.41 × 10^17^, 5.03 × 10^12^
Dichotomous Status-Liberal	Table 6	0.61 (0.494, 0.715)	1.56 × 10^10^ (2.19 × 10^8^, 1.11 × 10^12^)	3.13 × 10^10^, 4.39 × 10^8^

**Table 6:** E-Value List.

Number	E-Value Estimate	E-Value Lower Bound
1	Infinity	1.10 × 10^71^
2	1.78 × 10 × 10^34^	4.19 × 10^29^
3	1.03 × 10 × 10^34^	2.64 × 10^29^
4	7.23 × 10 × 10^20^	1.71 × 10^17^
5	1.41 × 10 × 10^17^	2.48 × 10^13^
6	9.02 × 10 × 10^13^	5.03 × 10^12^
7	7.25 × 10 × 10^13^	3.51 × 10^11^
8	1.75 × 10 × 10^13^	6.14 × 10^10^
9	2.89 × 10 × 10^12^	5.62 × 10^9^
10	6.93 × 10^11^	1.07 × 10^9^
11	3.59 × 10^11^	6.54 × 10^8^
12	3.13 × 10^10^	4.39 × 10^8^
13	1.57 × 10^10^	2.13 × 10^8^
14	1.49 × 10^9^	9.31 × 10^5^
15	2.75 × 10^7^	7.99 × 10^3^
16	1.12 × 10^7^	2.95 × 10^3^
17	1.08 × 10^6^	995.06
18	3.82 × 10^5^	654.55
19	2.08 × 10^5^	367.89
20	1.13 × 10^5^	137.45
21	3.89 × 10^3^	122.3
22	3.68 × 10^3^	61.76
23	3.09 × 10^3^	51.69
24	2.75 × 10^3^	36.41
25	2.55 × 10^3^	36.19
26	2.34 × 10^3^	30.32
27	1.95 × 10^3^	20.79
28	1.56 × 10^3^	13.02
29	534.51	11.5
30	331.35	11.23
31	303.98	10.92
32	1.53 × 10^2^	9.24
33	150.36	7
34	11.41	5.49
35	4.21	2.83
36	4.05	2.56
37	3.56	2.39
38	3.14	2.27
39	3.01	2.25
40	2.99	2.14
41	2.81	2
42	2.79	1.99
43	2.73	1.96
44	2.6	1.9
45	2.53	1.84
46	2.5	1.82
47	1.99	1.61
48	1.9	1.56
49	1.64	1.47
50	1.6	1.29
51	1.6	1.14
52	1.33	1.11
53	1.04	1.02
54	1.014	1.013
55	1.014	1.013
56	1.013	1.011
57	1.012	1.01
58	1.011	1.01
59	1.011	1.01
60	1.01	1.009
61	1.0087	1.0086
62	1.008	1.0079

**Table 7:** Multivariable Linear Regression of Cannabis Legal Status. With ETOPFA-Corrected DSR as the Dependent Variable.

Model and Term	Term Parameters		Model Parameters	
	Estimate (C.I.)	*p*-Value	Parameter	Value
				
**Legal Status**				
				
***Additive Cannabis***				
*lm(ETOPFA.Corrected.DSR~Cigarettes+Alcohol.Abuse+Cannabis.Exposure+Analgesics+Cocaine+4_Races+ Median.HH.Income+Status, weights = sw)*				
Median Household Income	0.56 (0.431, 0.682)	1.19 × 10^−16^	Adj R Squ.	0.7535
Hispanic	0.43 (0.291, 0.562)	1.63 × 10^−9^	F	90.14
NHBlack	0.4 (0.269, 0.522)	2.42 × 10^−9^	dF	12, 338
NHWhite	0.28 (0.135, 0.428)	1.85 × 10^−4^	S.D.	0.0356
Status-Decriminalised	0.28 (0.113, 0.44)	9.89 × 10^−4^	P	1.52 × 10^−97^
Analgesics	0.23 (0.128, 0.323)	7.92 × 10^−6^		
Cannabis.Exposure	0.2 (0.0917, 0.301)	2.62 × 10^−4^		
Alcoholism	0.18 (0.106, 0.254)	2.47 × 10^−6^		
Cigarettes	0.17 (0.0734, 0.275)	7.49 × 10^−4^		
Status-Medical	−0.11 (−0.308, 0.098)	0.3090		
Status-Legal	−0.52 (−56.8, 55.8)	0.9850		
Cocaine	−0.53 (−0.624, −0.439)	2.95 × 10^−25^		
				
***Additive Cannabinoids***				
*lm(ETOPFA.Corrected.DSR~Cigarettes+Alcohol.Abuse+THC.Exposure+Cannabigerol.Exposure+Analgesics+Cocaine+4_Races+ Median.HH.Income+Status, weights = sw)*				
Median Household Income	0.42 (0.285, 0.548)	1.55 × 10^−9^	Adj R Squ.	0.7535
Status-Decriminalised	0.27 (0.107, 0.435)	0.0013	F	90.14
NHBlack	0.22 (0.13, 0.316)	3.44 × 10^−6^	dF	12, 338
THC.Exposure	0.22 (0.115, 0.326)	5.22 × 10^−5^	S.D.	0.0356
Hispanic	0.21 (0.113, 0.309)	3.04 × 10^−5^	P	1.52 × 10^−97^
Alcoholism	0.21 (0.136, 0.28)	3.13 × 10^−8^		
Analgesics	0.2 (0.0999, 0.297)	9.05 × 10^−5^		
Cigarettes	0.13 (0.0272, 0.238)	0.0138		
Status-Medical	−0.05 (−0.248, 0.157)	0.6570		
Status-Legal	−0.42 (−57, 56.1)	0.9880		
Cocaine	−0.43 (−0.518, −0.342)	1.64 × 10^−19^		
				
***Interactive Cannabinoids***				
lm(ETOPFA.Corrected.DSR~Cigarettes*Alcohol.Abuse*THC.Exposure*Cannabigerol.Exposure+Analgesics+Cocaine+4_Races+ Median.HH.Income+Status, weights = sw)				
NHWhite: NHBlack: Hispanic: Asian	27.8 (24, 31.7)	1.15 × 10^−35^	Adj R Squ.	0.9285
NHBlack: Hispanic: Asian	23.6 (20.7, 26.5)	1.98 × 10^−43^	F	152.6
NHWhite: NHBlack: Asian	14.2 (12, 16.4)	4.26 × 10^−30^	dF	30, 320
NHWhite: Hispanic: Asian	14 (11.6, 16.3)	1.94 × 10^−26^	S.D.	0.0358
NHBlack: Asian	12.3 (10.4, 14.1)	1.39 × 10^−32^	P	1.69 × 10^−170^
Hispanic: Asian	9.64 (7.77, 11.5)	3.33 × 10^−21^		
NHWhite: Asian	8.78 (7.25, 10.3)	4.03 × 10^−25^		
NHWhite: NHBlack: Hispanic	7.51 (6.39, 8.63)	4.71 × 10^−32^		
NHBlack: Hispanic	7.03 (6.13, 7.93)	2.47 × 10^−40^		
Asian	5.54 (4.52, 6.56)	6.96 × 10^−23^		
NHBlack	3.48 (2.98, 3.98)	1.44 × 10^−33^		
NHWhite: NHBlack	3.14 (2.55, 3.72)	1.54 × 10^−22^		
NHWhite: Hispanic	2.76 (2.21, 3.31)	2.84 × 10^−20^		
Hispanic	2.41 (1.93, 2.88)	1.17 × 10^−20^		
NHWhite	1.76 (1.46, 2.06)	1.04 × 10^−25^		
Status-Medical	1.65 (1.42, 1.88)	3.11 × 10^−36^		
Cigarettes: Alcoholism: THC.Exposure	0.66 (0.546, 0.772)	9.79 × 10^−26^		
Cigarettes: Alcoholism: THC.Exposure: CBG.Exposure	0.63 (0.458, 0.8)	3.31 × 10^−12^		
CBG.Exposure	0.59 (0.424, 0.759)	2.13 × 10^−11^		
Cigarettes: CBG.Exposure	0.55 (0.151, 0.943)	6.94 × 10^−3^		
Cigarettes: THC.Exposure: CBG.Exposure	0.33 (0.0888, 0.568)	7.37 × 10^−3^		
Analgesics	0.3 (0.216, 0.381)	6.13 × 10^−12^		
Median Household Income	0.23 (0.0867, 0.377)	1.83 × 10^−3^		
Status-Decriminalised	0.23 (0.121, 0.34)	4.26 × 10^−5^		
Cocaine	0.28 (0.384, 0.181)	9.12 × 10^−8^		
Alcoholism: THC.Exposure: CBG.Exposure	−0.38 (−0.513, −0.251)	2.17 × 10^−8^		
Cigarettes	−0.51 (−0.672, −0.342)	4.33 × 10^−9^		
Cigarettes: THC.Exposure	−0.72 (−1.02, −0.421)	3.31 × 10^−6^		
Alcoholism: CBG.Exposure	−0.92 (−1.07, −0.769)	2.71 × 10^−28^		
Status-Legal	−1.04 (−31.4, 29.3)	0.9460		
**Dichotomous Legal Status**				
				
***Additive Cannabis***				
*lm(ETOPFA.Corrected.DSR~Cigarettes+Alcohol.Abuse+Cannabis.Exposure+Analgesics+Cocaine+4_Races+ Median.HH.Income+ Dichotomized.Status, weights = sw)*				
Median Household Income	0.87 (0.666, 1.07)	6.37 × 10^−16^	Adj R Squ.	0.7519
Dichotomous Status-Liberal	0.39 (0.212, 0.567)	2.05 × 10^−5^	F	107.1
THC.Exposure	0.38 (0.0965, 0.653)	0.0085	dF	10, 340
NHBlack	0.33 (0.212, 0.457)	1.52 × 10^−7^	S.D.	0.0357
Analgesics	0.27 (0.149, 0.383)	1.06 × 10^−5^	P	1.08 × 10^−98^
Alcoholism	0.26 (0.158, 0.357)	5.52 × 10^−7^		
Hispanic	0.24 (0.0851, 0.392)	0.0024		
Cigarettes	0.21 (0.0624, 0.348)	0.0050		
Cocaine	−0.3 (−0.435, −0.156)	3.99 × 10^−5^		
Asian	−0.49 (−0.827, −0.144)	0.0055		
CBG.Exposure	−0.49 (−0.813, −0.174)	0.0026		
				
***Additive Cannabinoids***				
*lm(ETOPFA.Corrected.DSR~Cigarettes+Alcohol.Abuse+THC.Exposure+Cannabigerol.Exposure+Analgesics+Cocaine+4_Races+ Median.HH.Income+Dichotomized.Status, weights = sw)*				
Median Household Income	0.53 (0.384, 0.682)	9.91 × 10^−12^	Adj R Squ.	0.7631
Cannabis.Exposure	0.44 (0.237, 0.65)	2.97 × 10^−5^	F	103.5
Hispanic	0.26 (0.145, 0.372)	1.09 × 10^−5^	dF	11, 339
Dichotomous Status-Liberal	0.26 (0.124, 0.387)	1.55 × 10^−4^	S.D.	0.0349
NHBlack	0.22 (0.132, 0.314)	2.09 × 10^−6^	P	2.92 × 10^−101^
Alcoholism	0.17 (0.0948, 0.242)	9.22 × 10^−6^		
Cigarettes	0.15 (0.0483, 0.26)	0.0044		
Analgesics	0.14 (0.0564, 0.23)	0.0013		
CBG.Exposure	−0.24 (−0.481, −0.00715)	0.0435		
Cocaine	−0.39 (−0.494, −0.287)	8.68 × 10^−13^		
Asian	−0.47 (−0.722, −0.215)	3.16 × 10^−4^		
***Interactive Cannabinoids***				
				
lm(ETOPFA.Corrected.DSR~Cigarettes*Alcohol.Abuse*THC.Exposure*Cannabigerol.Exposure+Analgesics+Cocaine+4_Races+ Median.HH.Income+Dichotomized.Status, weights = sw)				
NHWhite: NHBlack: Hispanic: Asian	26.8 (21.9, 31.6)	1.19 × 10^−23^	Adj R Squ.	0.8934
NHBlack: Hispanic: Asian	20 (16.5, 23.5)	3.31 × 10^−25^	F	109.7
NHWhite: Hispanic: Asian	15.1 (12.2, 18.1)	7.40 × 10^−21^	dF	27, 323
NHWhite: NHBlack: Asian	14 (11.2, 16.8)	2.64 × 10^−20^	S.D.	0.0234
NHBlack: Asian	11 (8.76, 13.3)	2.27 × 10^−19^	P	2.37 × 10^−145^
Hispanic: Asian	10.8 (8.46, 13.1)	8.68 × 10^−18^		
NHWhite: Asian	9.09 (7.14, 11)	5.81 × 10^−18^		
NHWhite: NHBlack: Hispanic	6.86 (5.49, 8.24)	3.87 × 10^−20^		
NHBlack: Hispanic	5.56 (4.49, 6.62)	1.07 × 10^−21^		
Asian	5.44 (4.12, 6.76)	1.12 × 10^−14^		
NHWhite: Hispanic	3.04 (2.37, 3.72)	4.41 × 10^−17^		
NHWhite: NHBlack	3 (2.27, 3.73)	1.46 × 10^−14^		
NHBlack	2.88 (2.28, 3.48)	9.10 × 10^−19^		
Hispanic	2.7 (2.14, 3.25)	4.92 × 10^−19^		
NHWhite	1.72 (1.34, 2.1)	4.80 × 10^−17^		
Cigarettes: Alcoholism: THC.Exposure: CBG.Exposure	1.22 (0.999, 1.43)	3.76 × 10^−24^		
Cigarettes: Alcoholism: THC.Exposure	1.18 (0.779, 1.59)	2.04 × 10^−8^		
THC.Exposure	1 (0.736, 1.27)	1.19 × 10^−12^		
Dichotomous Status-Liberal	0.61 (0.494, 0.715)	2.71 × 10^−23^		
Analgesics	0.35 (0.25, 0.443)	8.75 × 10^−12^		
Cigarettes: Alcoholism	0.29 (0.00514, 0.571)	4.60 × 10^−2^		
Median Household Income	0.25 (0.0671, 0.438)	7.76 × 10^−3^		
Cocaine	−0.34 (−0.444, −0.242)	9.40 × 10^−11^		
Alcoholism: THC.Exposure: CBG.Exposure	−0.66 (−0.876, −0.451)	2.51 × 10^−9^		
Alcoholism: CBG.Exposure	−0.92 (−1.15, −0.689)	4.18 × 10^−14^		
Cigarettes	−1.03 (−1.34, −0.714)	3.49 × 10^−10^		
Cigarettes: THC.Exposure	−1.03 (−1.36, −0.698)	3.09 × 10^−9^		

**Table 8:** Summary of Hill Criteria.

Hill Criterion	Supporting Evidence
	
Strength of Association	Yes. Hawaii, Colorado, Europe, USA, Australia, Canada (Refs. [3,15,17,21,22,23])
Consistency Amongst Studies	Yes. Hawaii, Colorado, Europe, USA, Australia, Canada (Refs. [3,15,17,21,22,23])
Specificity	Yes. Hawaii, Colorado, Europe, USA, Australia, Canada (Refs. [3,15,17,21,22,23])
Temporality	Yes. Hawaii, Colorado, Europe, USA, Australia, Canada (Refs. [3,15,17,21,22,23])
Coherence with Known Data	Yes. Hawaii, Colorado, Europe, USA, Australia, Canada (Refs. [3,15,17,21,22,23])
Biological Plausibility	Yes. Linked with both micronucleus formation and chromosomal mis-segregation. Also damage to eggs and sperm. Also many epigenetic mechanisms. See mechanistic discussion and also Ref. [15]
Dose–response Relationships	Yes. Hawaii, Colorado, Europe, USA, Australia, Canada (Refs. [3,15,17,21,22,23])
Analogy with Similar Situations Elsewhere	Yes. Hawaii, Colorado, Europe, USA, Australia, Canada (Refs. [3,15,17,21,22,23])
Experimental Confirmation	Yes. Linked with both micronucleus formation and chromosomal mis-segregation. See mechanistic discussion.

## Data Availability

All data generated or analysed during this study are included in this published article and its supplementary information files. Data along with the relevant R code has been made publicly available on the Mendeley Database Repository and can be accessed from this URL: https://data.mendeley.com/datasets/tn46tdhc4c/2 (accessed 13 October 2022).

## Supplementary Materials

The following supporting information can be downloaded at: https://www.mdpi.com/article/10.3390/ijerph192013340/s1, Table S1: Quinquennial Period; Table S2: Western Australian Downs Syndrome Rates 1980–2015; Table S3: Cannabis Use Quintiles; Table S4: Temporal and Quintile Bivariate Effects; Table S5: Panel Regression; Table S6: Kriged Data; Table S7: Mixed Effects models Including Ethnic Cannabis Exposure; Table S8: Linear Regression of Mulitvariable Adjustment for Cannabis Legal Status Dependent Variable is the Down Syndrome rate; Figure S1: Map graph of the logarithm of the Down syndrome rate corrected for estimates of the early termination of pregnancy for anomaly (ETOPFA) rate; Figure S2: Down syndrome rate across USA over time as a panelled and facetted scatterplot in alphabetical order; Figure S3: Down syndrome rate across USA over time as a geospatial panelled geofacetted scatterplot with each state in approximately its correct spatial location; Figure S4: ETOPFA-corrected Down syndrome rate across USA over time as a geospatial panelled geofacetted scatterplot with each state in approximately its correct spatial location; Figure S5: (A) Near daily cannabis use in pregnancy in NSDUH survey and (B) cannabis exposure in first trimester; Figure S6: Scatterplot of the effect of registry case ascertainment on the reported Down syndrome case rate; Figure S7: Corrgram correlogram of the Down syndrome rate by selected drug exposures and maternal ages; Figure S8: Corrgram correlogram of the Down syndrome rate by estimates of selected cannabinoid exposures; Figure S9: Corrgram correlogram of the Down syndrome rate by selected ethnic prevalences; Figure S10: Map graph of the logarithm of the kriged Down syndrome rate; Figure S11: (A) edited and (B) final interstate geospatial links which comprise the sparse spatial weights matrix used in all geospatial regressions.

## References

[1] Hickey F., Hickey E., Summar K.L. (2012). Medical update for children with Down syndrome for the pediatrician and family practitioner. Adv. Pediatr..

[2] Birth Defects: Facts about Down Syndrome. https://www.cdc.gov/ncbddd/birthdefects/downsyndrome.html.

[3] Birger Y., Shiloh R., Izraeli S. (2019). Mechanisms of Leukemia Evolution: Lessons from a Congenital Syndrome. Cancer Cell.

[4] Labuhn M., Perkins K., Matzk S., Varghese L., Garnett C., Papaemmanuil E., Metzner M., Kennedy A., Amstislavskiy V., Risch T. (2019). Mechanisms of Progression of Myeloid Preleukemia to Transformed Myeloid Leukemia in Children with Down Syndrome. Cancer Cell.

[5] McCantz-Katz E. (2018). Annual Report Snippets, NSDUH, SAMHSA, USA DHHS—Selected Streamlined Trends.

[6] Forrester M.B., Merz R.D. (2007). Risk of selected birth defects with prenatal illicit drug use, Hawaii, 1986–2002. J. Toxicol. Environ. Health.

[7] Public Health Agency of Canada, Health Canada (2013). Congenital Anomalies in Canada. A Perinatal Health Surveillance Report.

[8] Reece A.S., Hulse G.K. (2020). Canadian Cannabis Consumption and Patterns of Congenital Anomalies: An Ecological Geospatial Analysis. J. Addict. Med..

[9] (2018). National Cannabis Survey, Second Quarter. https://www.facebook.com/StatisticsCanada/posts/1636405843137586:0.

[10] Canada S., Statistics Canada (2019). Third Quarter National Cannabis Survey, Canada.

[11] Environment CDoPHat, Colorado: Department of Public Health and the Environment (2018). Colorado Responds to Children with Special Needs-Birth Defect Data, Colorado. Birth Defect Data-Colorado Register of Congenital Surveillance Network.

[12] Reece A.S., Hulse G.K. (2019). Cannabis Teratology Explains Current Patterns of Coloradan Congenital Defects: The Contribution of Increased Cannabinoid Exposure to Rising Teratological Trends. Clin. Pediatr..

[13] Health Q., Queensland Maternal and Perinatal Quality Council (2018). Queensland Mothers and Babies 2014 and 2015. Brisbane: Queensland Health.

[14] Reece A.S., Hulse G.K. (2022). Cannabinoid- and Substance- Relationships of European Congenital Anomaly Patterns: A Space-Time Panel Regression and Causal Inferential Study. Environ. Epigenetics.

[15] Reece A.S., Hulse G.K. (2022). Cannabis- and Substance- Related Epidemiological Patterns of Chromosomal Congenital Anomalies in Europe: Geospatiotemporal and Causal Inferential Study. Int. J. Environ. Res. Public Health.

[16] Colorado Department of Public Health and Environment (2020). THC Concentration in Colorado Marijuana.

[17] Reece A.S., Hulse G.K. (2021). Epidemiological Overview of Multidimensional Chromosomal and Genome Toxicity of Cannabis Exposure in Congenital Anomalies and Cancer Development. Sci. Rep..

[18] Reece A.S., Hulse G.K., Preedy V., Patel V., London U.K. (2022). Cannabinoid Genotoxicity and Congenital Anomalies: A Convergent Synthesis of European and USA Datasets. Cannabis, Cannabinoids and Endocannabinoids.

[19] Reece A.S., Hulse G.K. (2022). European Epidemiological Patterns of Cannabis- and Substance- Related Congenital Cardiovascular Anomalies: Geospatiotemporal and Causal Inferential Study. Environ. Epigenetics.

[20] Reece A.S., Hulse G.K. (2020). Cannabis in Pregnancy—Rejoinder, Exposition and Cautionary Tales. Psychiatr. Times.

[21] NBDPN (2018). National Birth Defects Prevention Network.

[22] Substance Abuse and Mental Health Services Administration (2018). National Survey of Drug Use and Health.

[23] Wonder C.D.C. Natality Information, Live Births. https://wonder.cdc.gov/natality.html.

[24] ElSohly M.A., Mehmedic Z., Foster S., Gon C., Chandra S., Church J.C. (2016). Changes in Cannabis Potency Over the Last 2 Decades (1995–2014): Analysis of Current Data in the United States. Biol. Psychiatry.

[25] ElSohly M.A., Ross S.A., Mehmedic Z., Arafat R., Yi B., Banahan B.F. (2000). Potency trends of delta9-THC and other cannabinoids in confiscated marijuana from 1980–1997. J. Forensic Sci..

[26] Women and Newborn Health Service, Department of Health, Government of Western Australia (2015). Western Australian Register of Developmental Anomalies 1980–2014.

[27] Bird T.M., Hobbs C.A., Cleves M.A., Tilford J.M., Robbins J.M. (2006). National rates of birth defects among hospitalized newborns. Birth Defects Res. A Clin. Mol. Teratol..

[28] Natoli J.L., Ackerman D.L., McDermott S., Edwards J.G. (2012). Prenatal diagnosis of Down syndrome: A systematic review of termination rates (1995–2011). Prenat. Diagn..

[29] Mansfield C., Hopfer S., Marteau T.M. (1999). Termination rates after prenatal diagnosis of Down syndrome, spina bifida, anencephaly, and Turner and Klinefelter syndromes: A systematic literature review. European Concerted Action: DADA (Decision-making After the Diagnosis of a fetal Abnormality). Prenat. Diagn..

[30] Wickham H., Averick M., Bryan J., Chang W., McGowan L.D., Francios R., Groelmund G., Hayes A., Henry L., Hester J. (2019). Welcome to the Tidyverse. J. Open Source Softw..

[31] Pebesma E. (2018). Simple Features for R: Standardized Support for Spatial Vector Data. R J..

[32] Geofacet ‘ggplot2’ Faceting Utilities for Geographical Data. https://CRAN.R-project.org/package=geofacet.

[33] Corrgram Plot a Correlogram. https://CRAN.R-project.org/package=corrgram.

[34] Wright K. (2013). Package ‘Corrgram’.

[35] Package ‘plm’. https://cran.r-project.org/web/packages/plm/plm.pdf.

[36] Lumley T. (2010). Complex Surveys: A Guide to Analysis Using R.

[37] Bivand R., Anselin L., Berke O., Bernat A., Carvalho M., Chun Y., Dormann C., Dray S., Halbersma R., Lewis-Koh N. (2007). The Spdep Package.

[38] Millo G. (2014). Maximum likelihood estimation of spatially and serially correlated panels with random effects. Comput. Stat. Data Anal..

[39] Millo G., Piras G. (2012). Splm: Spatial Panel Data Models in R. J. Stastistical Softw..

[40] Broom. Convert Statistical Objects into Tidy Tibbles. https://CRAN.R-project.org/package=broom.

[41] Broom.mixed. Tidying Methods for Mixed Models. http://github.com/bbolker/broom.mixed.

[42] Kapoor M., Kelejian H.H., Prucha I.R. (2007). Panel Data Models with Spatially Correlated Error Components. J. Econom..

[43] Millo G., Piras G. (2018). Package ‘splm’.

[44] Croissant Y., Millo G. (2019). Panel Data Econometrics with R.

[45] Van der Wal W.M., Geskus R.B. (2011). Ipw: An R Package for Inverse Probabilty Weighting. J. Stat. Softw..

[46] Pinheiro J., Bates D., DebRoy S., Sarkar D., Team R.C. (2020). Nlme: Linear and Nonlinear Mixed Effects Models.

[47] Package ‘Evalue’ Online Document. https://cran.r-project.org/web/packages/EValue/EValue.pdf.

[48] VanderWeele T.J., Ding P., Mathur M. (2019). Technical Considerations in the Use of the E-Value. J. Causal Inference.

[49] Wang J., Yuan W., Li M.D. (2011). Genes and pathways co-associated with the exposure to multiple drugs of abuse, including alcohol, amphetamine/methamphetamine, cocaine, marijuana, morphine, and/or nicotine: A review of proteomics analyses. Mol. Neurobiol..

[50] Reece A.S., Hulse G.K. (2016). Chromothripsis and epigenomics complete causality criteria for cannabis- and addiction-connected carcinogenicity, congenital toxicity and heritable genotoxicity. Mutat. Res./Fundam. Mol. Mech. Mutagen..

[51] Hembree W.C., Nahas G.G., Zeidenberg P., Huang H.F.S., Nahas G.G., Sutin K.M., Harvey D.J., Agurell S. (1999). Changes in human spermatozoa associated with high dose marihuana smoking.

[52] Zimmerman A.M., Zimmerman S., Braude M.C., Zimmerman A.M. (1987). Cytogenetic Studies of Cannabinoid Effects. Genetic and Perinatal Effects of Abused Substances.

[53] Zimmerman A.M., Zimmerman S., Raj A.Y., Nahas G.G., Sutin K.M., Harvey D.J., Agurell S., Totowa N.J. (1999). Effects of Cannabinoids on spermatogenesis in mice. Marihuana and Medicine.

[54] Zimmerman S., Zimmerman A.M. (1990). Genetic effects of marijuana. Int. J. Addict..

[55] Morishima A. (1984). Effects of cannabis and natural cannabinoids on chromosomes and ova. NIDA Res. Monogr..

[56] Allen E.G., Freeman S.B., Druschel C., Hobbs C.A., O’Leary L.A., Romitti P.A., Royle M.H., Torfs C.P., Sherman S.L. (2009). Maternal age and risk for trisomy 21 assessed by the origin of chromosome nondisjunction: A report from the Atlanta and National Down Syndrome Projects. Hum. Genet..

[57] Ghosh S., Feingold E., Dey S.K. (2009). Etiology of Down syndrome: Evidence for consistent association among altered meiotic recombination, nondisjunction, and maternal age across populations. Am J Med. Genet A.

[58] Vranekovic J., Bozovic I.B., Grubic Z., Wagner J., Pavlinic D., Dahoun S., Bena F., Culic V., Brajenovic-Milic B. (2012). Down syndrome: Parental origin, recombination, and maternal age. Genet Test Mol Biomarkers.

[59] Murphy S.K., Itchon-Ramos N., Visco Z., Huang Z., Grenier C., Schrott R., Acharya K., Boudreau M.H., Price T.M., Raburn D.J. (2018). Cannabinoid exposure and altered DNA methylation in rat and human sperm. Epigenetics.

[60] Schrott R., Acharya K., Itchon-Ramos N., Hawkey A.B., Pippen E., Mitchell J.T., Kollins S.H., Levin E.D., Murphy S.K. (2019). Cannabis use is associated with potentially heritable widespread changes in autism candidate gene DLGAP2 DNA methylation in sperm. Epigenetics.

[61] Almouzni G., Altucci L., Amati B., Ashley N., Baulcombe D., Beaujean N., Bock C., Bongcam-Rudloff E., Bousquet J., Braun S. (2014). Relationship between genome and epigenome--challenges and requirements for future research. BMC Genom..

[62] Vilne B., Schunkert H. (2018). Integrating Genes Affecting Coronary Artery Disease in Functional Networks by Multi-OMICs Approach. Front. Cardiovasc. Med..

[63] Schrott R., Murphy S.K., Modliszewski J.L., King D.E., Hill B., Itchon-Ramos N., Raburn D., Price T., Levin E.D., Vandrey R. (2021). Refraining from use diminishes cannabis-associated epigenetic changes in human sperm. Environ. Epigenetics.

[64] Reece A.S., Hulse G.K. (2020). Broad Spectrum epidemiological contribution of cannabis and other substances to the teratological profile of northern New South Wales: Geospatial and causal inference analysis. BMC Pharmacol. Toxicol..

[65] Reece A.S., Hulse G.K. (2022). Epigenomic and Other Evidence for Cannabis-Induced Aging Contextualized in a Synthetic Epidemiologic Overview of Cannabinoid-Related Teratogenesis and Cannabinoid-Related Carcinogenesis. Mendeley Data.

[66] Reece A.S., Hulse G.K. (2022). Cannabis, Cannabidiol, Cannabinoids and Multigenerational Policy. Engineering.

[67] Reece A.S., Hulse G.K. Extending the “Paracentral Dogma” of biology with the metabolome: Implications for understanding genomic-glycomic-metabolic-epigenomic synchronization. Engineering.

[68] Causal Inference in Statistics A Gentle Introduction. http://bayes.cs.ucla.edu/jsm-august2016-bw.pdf.

[69] VanderWeele T.J., Ding P. (2017). Sensitivity Analysis in Observational Research: Introducing the E-Value. Ann. Intern. Med..

[70] Pearl J., Mackenzie D. (2018). The Book of Why. The New Science of Cause and Effect.

[71] Hill A.B. (1965). The Environment and Disease: Association or Causation?. Proc. R Soc. Med..

[72] Cozens D.D., Nahas G.G., Harvey D., Nahas G.G., Sutin K.M., Harvey D.J., Agurell S. (1999). Prenatal Exposure to Cannabis and Fetal Development. Marijuana in Medicine.

[73] Tahir S.K., Zimmerman A.M. (1991). Influence of marihuana on cellular structures and biochemical activities. Pharmacol. Biochem. Behav..

[74] Russo C., Ferk F., Mišík M., Ropek N., Nersesyan A., Mejri D., Holzmann K., Lavorgna M., Isidori M., Knasmüller S. (2019). Low doses of widely consumed cannabinoids (cannabidiol and cannabidivarin) cause DNA damage and chromosomal aberrations in human-derived cells. Arch. Toxicol..

[75] (1976). McClean DK, Zimmerman AM: Action of delta 9-tetrahydrocannabinol on cell division and macromolecular synthesis in division-synchronized protozoa. Pharmacology.

[76] Parker S.J., Zuckerman B.S., Zimmermann A.M., Nahas G.G., Sutin K.M., Harvey D.J., Agurell S. (1999). The Effects of Maternal Marijuana Use During Pregnancy on Fetal Growth. Marijuana in Medicine.

[77] Tahir S.K., Trogadis J.E., Stevens J.K., Zimmerman A.M. (1992). Cytoskeletal organization following cannabinoid treatment in undifferentiated and differentiated PC12 cells. Biochem. Cell Biol..

[78] Thomas J., Tilak S., Zimmerman S., Zimmerman A.M. (1984). Action of delta 9-tetrahydrocannabinol on the pool of acid soluble nucleotides. Cytobios.

[79] Zimmerman A.M., Raj A.Y. (1980). Influence of cannabinoids on somatic cells in vivo. Pharmacology.

[80] Cozens D.D., Clark R., Palmer A.K., Hardy N., Nahas G.G., Harvey D.J. (1978). The effect of a crude marihuana extract on embryonic and foetal development of the rabbit. Adv. Biosci..

[81] Huang H.F.S., Nahas G.G., Hembree W.C., Nahas G.G., Sutin K.M., Harvey D.J., Agurell S. (1999). Effects of Marijuana Inhalation on Spermatogenesis of the Rat. Marijuana in Medicine.

[82] Morishima A., Henrich R.T., Jayaraman J., Nahas G.G. (1978). Hypoploid metaphases in cultured lymphocytes of marihuana smokers. Adv. Biosci..

[83] Nahas G.G., Morishima A., Desoize B. (1977). Effects of cannabinoids on macromolecular synthesis and replication of cultured lymphocytes. Fed. Proc..

[84] Ing G.M., Olman C.L., Oyd J.R. (1962). Drug-Induced (Thalidomide) Malformations. Can. Med. Assoc. J..

[85] Kizaki M., Hashimoto Y. (2008). New tubulin polymerization inhibitor derived from thalidomide: Implications for anti-myeloma therapy. Curr. Med. Chem..

[86] Iguchi T., Yachide-Noguchi T., Hashimoto Y., Nakazato S., Sagawa M., Ikeda Y., Kizaki M. (2008). Novel tubulin-polymerization inhibitor derived from thalidomide directly induces apoptosis in human multiple myeloma cells: Possible anti-myeloma mechanism of thalidomide. Int. J. Mol. Med..

[87] Tseng S., Pak G., Washenik K., Pomeranz M.K., Shupack J.L. (1996). Rediscovering thalidomide: A review of its mechanism of action, side effects, and potential uses. J. Am. Acad. Dermatol..

[88] Therapontos C., Erskine L., Gardner E.R., Figg W.D., Vargesson N. (2009). Thalidomide induces limb defects by preventing angiogenic outgrowth during early limb formation. Proc. Natl. Acad. Sci. USA.

[89] Vargesson N. (2009). Thalidomide-induced limb defects: Resolving a 50-year-old puzzle. Bioessays.

[90] Vargesson N. (2015). Thalidomide-induced teratogenesis: History and mechanisms. Birth Defects Res. Part C: Embryo Today: Rev..

[91] Khalil A., Tanos R., El-Hachem N., Kurban M., Bouvagnet P., Bitar F., Nemer G. (2017). A HAND to TBX5 Explains the Link Between Thalidomide and Cardiac Diseases. Sci. Rep..

[92] McBride W. (2004). Health of thalidomide victims and their progeny. Lancet.

[93] Rehman W., Arfons L.M., Lazarus H.M. (2011). The rise, fall and subsequent triumph of thalidomide: Lessons learned in drug development. Ther. Adv. Hematol..

[94] Franks M.E., Macpherson G.R., Figg W.D. (2004). Thalidomide. Lancet.

[95] Linz W. (1962). Thalidomide and Congenital Abnormalities. Lancet.

[96] Melchert M., List A. (2007). The thalidomide saga. Int. J. Biochem. Cell Biol..

[97] Reece A.S., Hulse G.K. (2020). Co-occurrence across time and space of drug- and cannabinoid- exposure and adverse mental health outcomes in the National Survey of Drug Use and Health: Combined geotemporospatial and causal inference analysis. BMC Public Health.

[98] Reece A.S., Hulse G.K. (2019). Epidemiological Associations of Various Substances and Multiple Cannabinoids with Autism in USA. Clin. Pediatr. Open Access.

[99] Reece A.S., Hulse G.K. (2019). Effect of Cannabis Legalization on US Autism Incidence and Medium Term Projections. Clin. Pediatr. Open Access.

[100] Reece A.S., Hulse G.K. (2019). Gastroschisis and Autism-Dual Canaries in the Californian Coalmine. JAMA Surg..

[101] Short T.D., Stallings E.B., Isenburg J., O’Leary L.A., Yazdy M.M., Bohm M.K., Ethen M., Chen X., Tran T., Fox D.J. (2019). Gastroschisis Trends and Ecologic Link to Opioid Prescription Rates—United States, 2006–2015. Morb. Mortal. Wkly. Rep..

