# Supplementary material for: Socioeconomic, Ethnocultural, Substance- and Cannabinoid-Related Epidemiology of Down Syndrome USA 1986–2016: Combined Geotemporospatial and Causal Inference Investigation
Source: Int J Environ Res Public Health. 2022 Oct 16;19(20):13340. doi: 10.3390/ijerph192013340 (PMC9602855; doi:10.3390/ijerph192013340)
Supplement: Supplementary file 1 [file ijerph-19-13340-s001.zip › ijerph-1874616-SM.pdf]

International Journal of
Environmental Research and Public Health

MDPI

Supplementary Table S1. Quinquennial Period

State	Quinquennial Period															
	1986-88	1989	1989-90	1991	1993-95	1995-96	1998-99	2003-07	2004-08	2005-09	2007-11	2008-12	2009-13	2010-2014	2011-15	2012-2016
Alabama							10.57									
Alaska						12.95	13.7	14.28	15.79	15.9			18.4	16.2	12.3	
Arizona	10.08	9.55	11.9	11.9		12.54		11.54	11.79	12.4	12.2	13	12.8	13	13.6	14.6
Arkansas	9.83			13.54	12.19	10.37	11.13	12.68	12.8	12	11.8	11.8	12.4	13.7	12.5	
California			11.18	13.08	12.26	12.87	11.73		14.11	14.1	14.4	14.3	14.4	15.7		15.9
Colorado			11.42	10.55	12.23	14.78	14.92	20.65	20.09	21.3	21.1	14.8	23.9	22.4	22.6	22.3
Connecticut					9.43	8.52		12.95		13.4						
Delaware							7.9			13.2	12.7	12.2	12.5	15	14.9	15.2
Florida							11.87	13.25	13.12	13	13.3	13.7	13.6	13.5	13.1	12.7
Georgia			10.03	10.68	10.97	12.71	12.45	14.5	16.42	16.7	21.4	21.8	20.9	19.6	16.3	13.4
Hawaii			10.78	7.55	14.6	19.09	16.05	12.32	12.7	8.9				15.3	14.2	15.3
Illinois			4.21	3.24	9.13	9.55	10.14	17.09	13.23	13	12.7	17.09	13.3	13.6	13.9	14.2
Indiana								10.86	11.22	12		11.2		8.7	11.6	13.4
Iowa	11.61	16.28	10.52			12.05	13.16	17.09	16.11	15.1	13.3	12	13.7	13.7	13.5	13.8
Kansas	6.07	3.88	7.21	8.32	7.44	6.54					11	11.5	11.2	10.6	10.8	10.5
Kentucky							4.25	13.01	12.41	12.6	8.9	8.2	10.8	13	15.3	14.6
Louisiana									16.71	12.9	11.7	9.9	9.5	11.5	12.4	12.1
Maine								11.56	12.91	12.7	13.7	13.5	12.2	12.8	12.7	11.5
Maryland	7.19	8.01	7.44			6.16	11.94	12.77	11.63	11.2	9.6	9.6	10.2	10	9.8	9.8
Massachusetts				8.26	8.36	11.71	8.04	12.45	12.35	12.7	14.8	17.5	19.7	22.7	25.6	25.6
Michigan							10.72		13.09	13.2	13.1	13.2	12.4	12.7	14.3	13.8
Minnesota										14.6	16.5	17	17.5	18.9	18.1	17.3
Mississippi								6.7	6.92	7.7	9.5	9.8	9.6	7.6	14.3	13.6
Missouri	8.09	10.35	12.24			10.58	13.68			16.2		13.1	13.6	13	13.7	14.8
Nebraska				11.94	12.6	12.86	11.53	16.66	14.95	15.4	19.2	18.3	18.7	18.2	15.7	18.2
Nevada					4.02	5.8			14.18	13.9	12.9	12.9	13.7	12.2	11.6	11.7
New Hampshire								15.05	11.37	10.7	10	9.3				
New Jersey			11.16	12.95	11.65	10.58	11.35	12.67	11.53	11.9	12.5	11.3	11.8	11.9	11.8	11.2
New Mexico					11.95	11.13	9.68	11.72			12	10.6	13.2	15	13.6	12.8
New York			10.87	10.69	9.58	8.88	9.87	12.6	12.37	12.3	12.3	12.3	12.4	14.2	13.3	13
North Carolina			8.05	9.67	9.76	12.05	12.02	12.67	12.34	12.1	12.5	12.6	12.7	13.3	13.1	13
North Dakota								8.82	9.09	8	10	11.4	11.1	11.1		
Ohio					4.58	4.87					11.2				10.1	14.5
Oklahoma			12.86	9.55	10.91	13.56	12.72	12.9	12.9	11.9	13.1	12.8	12.8	13.1	12	11.1
Oregon												16.5	18.9	18.9	18.6	18.2
Puerto Rico								13.34	14.4	15	14.7	14.3	13.2	13	11.2	11

Rhode Island			10.92			9.52	10.66	13.7	15.8	17	17.3	15.1	12.8	14.8
South Carolina			2.85	2.13				9.9	8.3	10.8	11.2	11.7	11.7	12.3
Tennessee		5.31	5.18			13.57	14.17	14.4	14.2	14.6	14.2	14.5	14.9	14.8
Texas				14.47	11.02	13.6	13.38	13.5	13.6	14.3	14.5	14.5	13.9	13.9
Utah			7.03	16.55	15.51	15.16	14.38	14.6	15.4	15.5	16.1	16.2	17.3	17.2
Vermont									13.4	14	10.9	10.5	10.6	9.1
Virginia	8.78	10.26	5.47	5.1	8.54		14.37	12.9	12.9	12.8	12.6	11.8	12	
Washington	12.69	7.7										13.1	14.8	14.6
West Virginia	5	3.28	5.67			8.53	8.29	7.3	7.2	8.3	8	7.7	6.8	6.2
Wisconsin	3.81	4.73	11.72	11.84	11.79		12.99	12.7	12.4	11.4	11.2	11.2	10.9	10.7

Supplementary Table S2. Western Australian Downs Syndrome Rates 1980-2015.

Year	Live.Births	ETOPFA	Total.T21	WA.T21.TOP.Rate	TopRt	FMaxTR
1980	1.0987	0.0000	1.0987	0.0000	0.0000	0.0000
1981	0.8537	0.1254	0.9791	0.1275	0.1275	0.1645
1982	1.1136	0.1184	1.2320	0.0941	0.0941	0.1215
1983	1.0850	0.1115	1.1965	0.0923	0.0923	0.1191
1984	0.9987	0.1910	1.1897	0.1581	0.1581	0.2041
1985	1.1504	0.3283	1.4787	0.2183	0.2183	0.2818
1986	1.1651	0.4079	1.5729	0.2496	0.2496	0.3222
1987	1.0283	0.3144	1.3427	0.2131	0.2131	0.2750
1988	1.0863	0.5670	1.6533	0.3323	0.3323	0.4289
1989	0.9783	0.2932	1.2716	0.2276	0.2276	0.2938
1990	1.1156	0.3368	1.4523	0.2135	0.2135	0.2756
1991	1.0437	0.4235	1.4673	0.2722	0.2722	0.3514
1992	1.2603	0.5247	1.7851	0.2834	0.2834	0.3658
1993	1.4625	0.5250	1.9875	0.2521	0.2521	0.3253
1994	0.6695	0.8570	1.5265	0.5557	0.5557	0.7173
1995	1.0231	1.1168	2.1399	0.5080	0.5080	0.6557
1996	1.0882	0.8358	1.9241	0.4141	0.4141	0.5345
1997	0.8433	1.0092	1.8525	0.5322	0.5322	0.6869
1998	0.7498	0.9517	1.7015	0.5301	0.5301	0.6842
1999	0.8149	1.0241	1.8390	0.5399	0.5399	0.6969
2000	1.2767	1.7022	2.9789	0.5606	0.5606	0.7236
2001	0.7289	1.3779	2.1068	0.6454	0.6454	0.8330
2002	1.1402	1.5080	2.6482	0.5430	0.5430	0.7008
2003	0.8232	1.4073	2.2304	0.6058	0.6058	0.7819
2004	1.0974	1.7032	2.8006	0.5800	0.5800	0.7487
2005	1.0400	1.8621	2.9021	0.6341	0.6341	0.8184
2006	0.8383	1.6893	2.5276	0.6539	0.6539	0.8440
2007	1.0044	1.6823	2.6868	0.6008	0.6008	0.7755
2008	0.7523	1.5095	2.2618	0.6224	0.6224	0.8033
2009	0.9761	1.9857	2.9618	0.6489	0.6489	0.8375
2010	0.9582	2.2352	3.1935	0.6997	0.6997	0.9031
2011	0.6527	2.2291	2.8819	0.7815	0.7815	1.0087

2012	0.7383	2.1802	2.9185	0.7531	0.7531	0.9720
2013	0.7200	2.0825	2.8024	0.7461	0.7461	0.9630
2014	0.5122	1.8075	2.3198	0.7748	0.7748	1.0000
2015	0.5122	1.8075	2.3198	0.7748	0.7748	1.0000

Supplementary Table S3. Cannabis Use Quintiles.

Rank Ordered Listing					
**State**	**Year**	**mrjmon**	**QuinCI**	**Quin**	**QuinCN**
Colorado	2015	0.171216	(0.146,0.171]	Quintile 5	(0.113,0.171]
Vermont	2015	0.150801	(0.146,0.171]	Quintile 5	(0.113,0.171]
Alaska	2015	0.147986	(0.146,0.171]	Quintile 5	(0.113,0.171]
Maine	2015	0.139832	(0.122,0.146]	Quintile 4	(0.113,0.171]
Rhode Island	2015	0.132805	(0.122,0.146]	Quintile 4	(0.113,0.171]
Oregon	2015	0.130385	(0.122,0.146]	Quintile 4	(0.113,0.171]
New Hampshire	2015	0.123054	(0.122,0.146]	Quintile 4	(0.113,0.171]
District of Columbia	2015	0.121021	(0.0968,0.122]	Quintile 3	(0.113,0.171]
Massachusetts	2015	0.119083	(0.0968,0.122]	Quintile 3	(0.113,0.171]
Washington	2015	0.114177	(0.0968,0.122]	Quintile 3	(0.113,0.171]
Montana	2015	0.113184	(0.0968,0.122]	Quintile 3	(0.0817,0.113]
New Mexico	2015	0.103081	(0.0968,0.122]	Quintile 3	(0.0817,0.113]
Michigan	2015	0.102894	(0.0968,0.122]	Quintile 3	(0.0817,0.113]
California	2015	0.098151	(0.0968,0.122]	Quintile 3	(0.0817,0.113]
New York	2015	0.097454	(0.0968,0.122]	Quintile 3	(0.0817,0.113]
Connecticut	2015	0.097221	(0.0968,0.122]	Quintile 3	(0.0817,0.113]
Maryland	2015	0.096645	(0.072,0.0968]	Quintile 2	(0.0817,0.113]
Indiana	2015	0.087862	(0.072,0.0968]	Quintile 2	(0.0817,0.113]
Arizona	2015	0.085918	(0.072,0.0968]	Quintile 2	(0.0817,0.113]
South Carolina	2015	0.082666	(0.072,0.0968]	Quintile 2	(0.0817,0.113]
Missouri	2015	0.081698	(0.072,0.0968]	Quintile 2	(0.0743,0.0817]
Hawaii	2015	0.081108	(0.072,0.0968]	Quintile 2	(0.0743,0.0817]
Delaware	2015	0.081095	(0.072,0.0968]	Quintile 2	(0.0743,0.0817]
Illinois	2015	0.080563	(0.072,0.0968]	Quintile 2	(0.0743,0.0817]
Georgia	2015	0.078868	(0.072,0.0968]	Quintile 2	(0.0743,0.0817]
Florida	2015	0.078732	(0.072,0.0968]	Quintile 2	(0.0743,0.0817]
Nevada	2015	0.078481	(0.072,0.0968]	Quintile 2	(0.0743,0.0817]
Ohio	2015	0.078167	(0.072,0.0968]	Quintile 2	(0.0743,0.0817]
Pennsylvania	2015	0.078005	(0.072,0.0968]	Quintile 2	(0.0743,0.0817]
Minnesota	2015	0.07738	(0.072,0.0968]	Quintile 2	(0.0743,0.0817]
North Carolina	2015	0.074308	(0.072,0.0968]	Quintile 2	(0.064,0.0743]
Kansas	2015	0.073762	(0.072,0.0968]	Quintile 2	(0.064,0.0743]
Arkansas	2015	0.072504	(0.072,0.0968]	Quintile 2	(0.064,0.0743]
Kentucky	2015	0.071327	[0.0472,0.072]	Quintile 1	(0.064,0.0743]
New Jersey	2015	0.070252	[0.0472,0.072]	Quintile 1	(0.064,0.0743]
West Virginia	2015	0.069969	[0.0472,0.072]	Quintile 1	(0.064,0.0743]
South Dakota	2015	0.069882	[0.0472,0.072]	Quintile 1	(0.064,0.0743]
Wisconsin	2015	0.068192	[0.0472,0.072]	Quintile 1	(0.064,0.0743]
Virginia	2015	0.067445	[0.0472,0.072]	Quintile 1	(0.064,0.0743]
Louisiana	2015	0.064257	[0.0472,0.072]	Quintile 1	(0.064,0.0743]
Nebraska	2015	0.063974	[0.0472,0.072]	Quintile 1	[0.0472,0.064]

Tennessee	2015	0.063807	[0.0472,0.072]	Quintile 1	[0.0472,0.064]
Oklahoma	2015	0.063326	[0.0472,0.072]	Quintile 1	[0.0472,0.064]
Idaho	2015	0.063242	[0.0472,0.072]	Quintile 1	[0.0472,0.064]
Wyoming	2015	0.062962	[0.0472,0.072]	Quintile 1	[0.0472,0.064]
Texas	2015	0.060034	[0.0472,0.072]	Quintile 1	[0.0472,0.064]
North Dakota	2015	0.059227	[0.0472,0.072]	Quintile 1	[0.0472,0.064]
Alabama	2015	0.053685	[0.0472,0.072]	Quintile 1	[0.0472,0.064]
Mississippi	2015	0.053619	[0.0472,0.072]	Quintile 1	[0.0472,0.064]
Utah	2015	0.051131	[0.0472,0.072]	Quintile 1	[0.0472,0.064]
Iowa	2015	0.047242	[0.0472,0.072]	Quintile 1	[0.0472,0.064]

Alphabetical Listing

State	Year	mrjmon	QuinCI	Quin	QuinCN
Alabama	2015	0.053685	[0.0472,0.072]	Quintile 1	[0.0472,0.064]
Alaska	2015	0.147986	(0.146,0.171]	Quintile 5	(0.113,0.171]
Arizona	2015	0.085918	(0.072,0.0968]	Quintile 2	(0.0817,0.113]
Arkansas	2015	0.072504	(0.072,0.0968]	Quintile 2	(0.064,0.0743]
California	2015	0.098151	(0.0968,0.122]	Quintile 3	(0.0817,0.113]
Colorado	2015	0.171216	(0.146,0.171]	Quintile 5	(0.113,0.171]
Connecticut	2015	0.097221	(0.0968,0.122]	Quintile 3	(0.0817,0.113]
Delaware	2015	0.081095	(0.072,0.0968]	Quintile 2	(0.0743,0.0817]
District of Columbia	2015	0.121021	(0.0968,0.122]	Quintile 3	(0.113,0.171]
Florida	2015	0.078732	(0.072,0.0968]	Quintile 2	(0.0743,0.0817]
Georgia	2015	0.078868	(0.072,0.0968]	Quintile 2	(0.0743,0.0817]
Hawaii	2015	0.081108	(0.072,0.0968]	Quintile 2	(0.0743,0.0817]
Idaho	2015	0.063242	[0.0472,0.072]	Quintile 1	[0.0472,0.064]
Illinois	2015	0.080563	(0.072,0.0968]	Quintile 2	(0.0743,0.0817]
Indiana	2015	0.087862	(0.072,0.0968]	Quintile 2	(0.0817,0.113]
Iowa	2015	0.047242	[0.0472,0.072]	Quintile 1	[0.0472,0.064]
Kansas	2015	0.073762	(0.072,0.0968]	Quintile 2	(0.064,0.0743]
Kentucky	2015	0.071327	[0.0472,0.072]	Quintile 1	(0.064,0.0743]
Louisiana	2015	0.064257	[0.0472,0.072]	Quintile 1	(0.064,0.0743]
Maine	2015	0.139832	(0.122,0.146]	Quintile 4	(0.113,0.171]
Maryland	2015	0.096645	(0.072,0.0968]	Quintile 2	(0.0817,0.113]
Massachusetts	2015	0.119083	(0.0968,0.122]	Quintile 3	(0.113,0.171]
Michigan	2015	0.102894	(0.0968,0.122]	Quintile 3	(0.0817,0.113]
Minnesota	2015	0.07738	(0.072,0.0968]	Quintile 2	(0.0743,0.0817]
Mississippi	2015	0.053619	[0.0472,0.072]	Quintile 1	[0.0472,0.064]
Missouri	2015	0.081698	(0.072,0.0968]	Quintile 2	(0.0743,0.0817]
Montana	2015	0.113184	(0.0968,0.122]	Quintile 3	(0.0817,0.113]
Nebraska	2015	0.063974	[0.0472,0.072]	Quintile 1	[0.0472,0.064]
Nevada	2015	0.078481	(0.072,0.0968]	Quintile 2	(0.0743,0.0817]
New Hampshire	2015	0.123054	(0.122,0.146]	Quintile 4	(0.113,0.171]
New Jersey	2015	0.070252	[0.0472,0.072]	Quintile 1	(0.064,0.0743]
New Mexico	2015	0.103081	(0.0968,0.122]	Quintile 3	(0.0817,0.113]
New York	2015	0.097454	(0.0968,0.122]	Quintile 3	(0.0817,0.113]
North Carolina	2015	0.074308	(0.072,0.0968]	Quintile 2	(0.064,0.0743]
North Dakota	2015	0.059227	[0.0472,0.072]	Quintile 1	[0.0472,0.064]
Ohio	2015	0.078167	(0.072,0.0968]	Quintile 2	(0.0743,0.0817]

Oklahoma	2015	0.063326	[0.0472,0.072]	Quintile 1	[0.0472,0.064]
Oregon	2015	0.130385	(0.122,0.146]	Quintile 4	(0.113,0.171]
Pennsylvania	2015	0.078005	(0.072,0.0968]	Quintile 2	(0.0743,0.0817]
Rhode Island	2015	0.132805	(0.122,0.146]	Quintile 4	(0.113,0.171]
South Carolina	2015	0.082666	(0.072,0.0968]	Quintile 2	(0.0817,0.113]
South Dakota	2015	0.069882	[0.0472,0.072]	Quintile 1	(0.064,0.0743]
Tennessee	2015	0.063807	[0.0472,0.072]	Quintile 1	[0.0472,0.064]
Texas	2015	0.060034	[0.0472,0.072]	Quintile 1	[0.0472,0.064]
Utah	2015	0.051131	[0.0472,0.072]	Quintile 1	[0.0472,0.064]
Vermont	2015	0.150801	(0.146,0.171]	Quintile 5	(0.113,0.171]
Virginia	2015	0.067445	[0.0472,0.072]	Quintile 1	(0.064,0.0743]
Washington	2015	0.114177	(0.0968,0.122]	Quintile 3	(0.113,0.171]
West Virginia	2015	0.069969	[0.0472,0.072]	Quintile 1	(0.064,0.0743]
Wisconsin	2015	0.068192	[0.0472,0.072]	Quintile 1	(0.064,0.0743]
Wyoming	2015	0.062962	[0.0472,0.072]	Quintile 1	[0.0472,0.064]

Supplementary Table S4. Temporal and Quintile Bivariate Effects.

Parameter	Model		Model Parameters			
	Estimate (C.I.)	Pr(>|t|)	R-Squared	F	dF	P
lm(Downs_Rate ~ Year)						
Year	0.21 (0.17, 0.25)	<2.2E-16	0.2096	115.3	1,430	<2.2E-16
lm(Downs_Rate ~ Cannabis_Monthly)						
Cannabis, Monthly	2.97 (1.91, 4.03)	8.2E-08	0.0806	30.11	1,331	8.2E-08
lm(Downs_Rate ~ Cannabis_Use_Quintile)						
Quintile 5	3.86 (2.45, 5.27)	1.2E-07	0.0772	10.01	4,427	9.4E-08
Quintile 4	1.74 (0.41, 3.07)	0.0112				
Quintile 3	1.28 (0.28, 2.28)	0.0122				
lm(Downs_Rate ~ Cannabis_Use_Dichotomized_Quintile)						
Highest Quintile	3.54 (2.19, 4.89)	4.4E-07	0.0555	26.34	1,430	4.4E-07
*lm(Downs_Rate ~ Year * Cannabis_Use_Quintile)*						
Quintile 5	0.0019 (0.001, 0.002)	7.9E-09	0.2762	33.9	5,426	<2.2E-16
Quintile 3	0.0007 (0.000, 0.001)	0.0015				
*lm(Downs_Rate ~ Year * Cannabis_Use_Dichotomized_Quintile)*						
Highest Quintile	3.35 (2.15, 4.55)	7.24E-08	0.2596	76.56	2,429	<2.2E-16

Supplementary Table S5. Panel Regression.

Instrumental ± Lagged Variables	Model				Model		
	Parameter	Estimate (C.I.)	P-Value	LogLik	Parameters	Value	P-Value

All Variables							
0 Lags							
spreml(ETOPFA_Adj_DS_Rate ~ cigmon * THC_Exposure * Cannabigerol_Exposure * abodalc + anlyr + cocyr + MHY + 5_Races + AdvMatlAge)							
THC__Exposure	Asian.Am	6.67 (3.2, 10.13)	0.0002	-1065.553	phi	0.2119	NA
CBG_Exposure	Cigarettes	2041.32 (702.49, 3380.14)	0.0028		psi	0.8193	NA
NHWhite_THC_Exposure	Cigarettes: Analgesics	645.08 (209.04, 1081.12)	0.0037		rho	0.7525	<2.0E-16
NHBlack_THC_Exposure	Analgesics	-72.67 (-125.14, -20.2)	0.0066		lambda	-0.3457	0.0002
NHAIAN_THC_Exposure	Cigarettes: THC.Exposure	9881.72 (1914.13, 17849.3)	0.0151				
Hispanic_THC_Exposure	Cigarettes: THC.Exposure: Cannabigerol.Exposure	2477.53 (468.35, 4486.72)	0.0157				
NHAsian_THC_Exposure	Cigarettes: THC.Exposure: Analgesics	3166.94 (554.84, 5779.04)	0.0175				
	Cigarettes: THC.Exposure: Cannabigerol.Exposure: Analgesics	796.37 (138.72, 1454.02)	0.0176				
	THC.Exposure	-2292.51 (-4254.45, -330.57)	0.0220				
	THC.Exposure: Analgesics	-738.53 (-1381.3, -95.75)	0.0243				
	THC.Exposure: Cannabigerol.Exposure	-559.68 (-1052.49, -66.86)	0.0260				
	THC.Exposure: Cannabigerol.Exposure: Analgesics	-180.8 (-342.07, -19.53)	0.0280				
	Cigarettes: Cannabigerol.Exposure	350.73 (29.16, 672.3)	0.0325				
	Cigarettes: Cannabigerol.Exposure: Analgesics	110.25 (5.14, 215.36)	0.0398				
	Cauc.Am	21.33 (0.68, 41.98)	0.0429				
2 Lags - Cannabinoids							
spreml(ETOPFA_Adj_DS_Rate ~ cigmon * THC_Exposure * Cannabigerol_Exposure * abodalc + anlyr + cocyr + MHY + 5_Races + AdvMatlAge)							
THC_Exposure, 0:2	Cigarettes: THC.Exposure: Cannabigerol.Exposure: Analgesics	1950.33 (826.32, 3074.34)	0.0007	-629.9304	phi	3.1E-05	0.0E+00
CBG_Exposure, 0:2	Cigarettes: THC.Exposure: Cannabigerol.Exposure	6076.82 (2538.81, 9614.83)	0.0008		psi	0.8095	<2.0E-16
NHWhite_Can_THCExposure, 0:2	THC.Exposure: Cannabigerol.Exposure: Analgesics	-458.76 (-728.03, -189.5)	0.0008		rho	-0.6038	2.18E-06
NHBlack_Can_THCExposure, 0:2	THC.Exposure: Cannabigerol.Exposure	-1428.07 (-2278.41, -577.73)	0.0010		lambda	0.7221	<2.0E-16
NHAIAN_Can_THCExposure, 0:2	Cigarettes: THC.Exposure: Analgesics	6976.96 (2808.73, 11145.19)	0.0010				

Hispanic_Can_THCExposure, 0:2	Cigarettes: THC.Exposure	21739.52 (8655.9, 34823.14)	0.0011				
NHAsian_Can_THCExposure, 0:2	THC.Exposure: Analgesics	-1616.88 (-2608.25, -625.51)	0.0014				
	THC.Exposure	-5020.66 (-8143.21, -1898.11)	0.0016				
	Cannabigerol.Exposure	-8.48 (-14.87, -2.09)	0.0093				
	Hispanic.Am	2.12 (0.13, 4.11)	0.0370				
4 Lags - Cannabinoids							
spreml(ETOPFA_Adj_DS_Rate ~ cigmon * THC_Exposure * Cannabigerol_Exposure * abodalc + anlyr + cocyr + MHY + 5_Races + AdvMatlAge)							
THC_Exposure, 0:4	Alcohol.Abuse	-467.4 (-690.25, -244.55)	3.9E-05	-233.6686	phi	1.61874	<2.0E-16
CBG_Exposure, 0:4	Cigarettes: THC.Exposure: Cannabigerol.Exposure	13554 (6435.08, 20672.92)	0.0002		psi	0.95012	<2.0E-16
NHWhite_Can_THCExposure, 0:4	Cigarettes: THC.Exposure: Cannabigerol.Exposure: Analgesics	4184.3 (1964.6, 6404)	0.0002		rho	0.267008	NA
NHBlack_Can_THCExposure, 0:4	Cannabigerol.Exposure	-68145 (-105449.68, -30840.32)	0.0003		lambda	-0.11837	NA
NHAIAN_Can_THCExposure, 0:4	THC.Exposure: Cannabigerol.Exposure	-3065.6 (-4747.67, -1383.53)	0.0004				
Hispanic_Can_THCExposure, 0:4	THC.Exposure: Cannabigerol.Exposure: Analgesics	-946.6 (-1472.66, -420.54)	0.0004				
NHAsian_Can_THCExposure, 0:4	Cannabigerol.Exposure: Analgesics	-20840 (-32513.96, -9166.04)	0.0005				
	Analgesics	-67542 (-105393.52, -29690.48)	0.0005				
	Cigarettes: Cannabigerol.Exposure	281220 (121093.88, 441346.12)	0.0006				
	Cigarettes	911500 (392335.2, 1430664.8)	0.0006				
	Cigarettes: Cannabigerol.Exposure: Analgesics	86662 (36562.44, 136761.56)	0.0007				
	Cigarettes: Analgesics	280890 (118435.4, 443344.6)	0.0007				
	THC.Exposure	56718 (19227.12, 94208.88)	0.0030				
	THC.Exposure: Analgesics	17300 (5555.48, 29044.52)	0.0039				
	Cigarettes: THC.Exposure	-230270 (-392346.32, -68193.68)	0.0054				
	Cigarettes: THC.Exposure: Analgesics	-70893 (-121608, -20178)	0.0061				

Asian.Am	8.10 (2.22, 13.98)	0.0076
Cauc.Am	44.00 (10.88, 77.12)	0.0094
Hispanic.Am	6.40 (1.11, 11.69)	0.0164
Median.HH.Income	-14.40 (-28.12, -0.68)	0.0394

Supplementary Table S6. Kriged Data.

State	2005	2006	2007	2009	2010	2011	2012	2013	2014
Alaska	14.3	15.8	15.9	**16.5**	**17.2**	**17.8**	18.4	16.2	12.3
Arizona	11.5	11.8	12.4	12.2	13.0	12.8	13.0	13.6	14.6
Arkansas	12.8	12.0	11.8	11.8	12.4	13.7	**13.1**	12.5	**12.5**
California	**14.1**	14.1	14.1	14.4	14.3	14.4	15.7	**15.8**	15.9
Colorado	20.7	20.1	21.3	21.1	14.8	23.9	22.4	22.6	22.3
Delaware	**13.7**	**13.7**	13.2	12.7	12.2	12.5	15.0	14.9	15.2
Florida	13.3	13.1	13.0	13.3	13.7	13.6	13.5	13.1	12.7
Georgia	14.5	16.4	16.7	21.4	21.8	20.9	19.6	16.3	13.4
Hawaii	12.3	12.7	8.9	**10.5**	**12.1**	**13.7**	15.3	14.2	15.3
Illinois	17.1	13.2	13.0	12.7	17.1	13.3	13.6	13.9	14.2
Indiana	10.9	11.2	12.0	**11.6**	11.2	**11.3**	**11.5**	11.6	13.4
Iowa	17.1	16.1	15.1	13.3	12.0	13.7	13.7	13.5	13.8
Kansas	**10.9**	**10.9**	10.9	11.0	11.5	11.2	10.6	10.8	10.5
Kentucky	13.0	12.4	12.6	8.9	8.2	10.8	13.0	15.3	14.6
Louisiana	**16.7**	16.7	12.9	11.7	9.9	9.5	11.5	12.4	12.1
Maine	11.6	12.9	12.7	13.7	13.5	12.2	12.8	12.7	11.5
Maryland	12.8	11.6	11.2	9.6	9.6	10.2	10.0	9.8	9.8
Massachusetts	12.5	12.4	12.7	14.8	17.5	19.7	22.7	25.6	25.6
Michigan	**13.1**	13.1	13.2	13.1	13.2	12.4	12.7	14.3	13.8
Minnesota	**17.1**	**17.1**	14.6	16.5	17.0	17.5	18.9	18.1	17.3
Mississippi	6.7	6.9	7.7	9.5	9.8	9.6	7.6	14.3	13.6
Missouri	**14.2**	**14.2**	16.2	**14.7**	13.1	13.6	13.0	13.7	14.8
Nebraska	16.7	15.0	15.4	19.2	18.3	18.7	18.2	53.3	18.2
Nevada	**14.2**	14.2	13.9	12.9	12.9	13.7	12.2	11.6	11.7
New Jersey	12.7	11.5	11.9	12.5	11.3	11.8	11.9	11.8	11.2
New Mexico	11.7	**11.8**	**11.9**	12.0	10.6	13.2	15.0	13.6	12.8
New York	12.6	12.4	12.3	12.3	12.3	12.4	14.2	13.3	13.0
North Carolina	12.7	12.3	12.1	12.5	12.6	12.7	13.3	13.1	13.0
North Dakota	8.8	9.1	8.0	10.0	11.4	11.1	11.1	**10.5**	**9.9**
Oklahoma	12.9	12.9	11.9	13.1	12.8	12.8	13.1	12.0	11.1
Rhode Island	9.5	10.7	13.7	15.8	17.0	17.3	##	12.8	14.8
South Carolina	**10.8**	**10.8**	9.9	8.3	10.8	11.2	11.7	11.7	12.3
Tennessee	13.6	14.2	14.4	14.2	14.6	14.2	14.5	14.9	14.8
Texas	13.6	13.4	13.5	13.6	14.3	14.5	14.5	13.9	13.9
Utah	15.2	14.4	14.6	15.4	15.5	16.1	16.2	17.3	17.2
Vermont	**11.4**	**11.4**	**12.4**	13.4	14.0	10.9	10.5	10.6	9.1
Virginia	**12.8**	14.4	12.9	12.9	12.8	12.6	11.8	12.0	**12.8**
West Virginia	8.5	8.3	7.3	7.2	8.3	8.0	7.7	6.8	6.2
Wisconsin	**13.0**	13.0	12.7	12.4	11.4	11.2	11.2	10.9	10.7

Supplementary Table S7. Mixed Effects models Including Ethnic Cannabis Exposure.

Parameter	Estimate (C.I.)	P-Value

**Additive Model**		
lme(Downs_Rate ~ Cigarettes+Alcohol.Abuse+THC.Exposure+Canabigerol.Exposure+Analgesics+Cocaine+4_Races+ Median.HH.Income+5_Races_THC_Exposure, random=~1|id, weights = ~ sw)		
Asian.THC.Exposure	0.62 (0.61, 0.64)	<0.0001
Cauc.THC.Exposure	4.87 (4.67, 5.08)	<0.0001
Cocaine	0.33 (0.31, 0.34)	<0.0001
Hispanic	0.70 (0.62, 0.78)	<0.0001
Alcohol.Abuse	6.62 (5.23, 8.02)	<0.0001
NH_Afric.Am	0.16 (0.12, 0.21)	<0.0001
NH_Cauc.Am	-2.56 (-3.21, -1.91)	<0.0001
Cigarettes	-1.67 (-1.98, -1.35)	<0.0001
Median.HH.Income	-0.27 (-0.3, -0.25)	<0.0001
Cannabigerol_Exposure	-2.10 (-2.2, -2.01)	<0.0001
Afric.THC.Exposure	-3.65 (-3.8, -3.49)	<0.0001
NH_Asian.Am	-1.34 (-1.38, -1.31)	<0.0001
**Model Interactive in Drugs**		
lme(Downs_Rate ~ Cigarettes*Alcohol.Abuse*THC.Exposure*Canabigerol.Exposure*Analgesics+Cocaine+ 4_Races+ Median.HH.Income+5_Races_THC_Exposure, random=~1|id, weights = ~ sw)		
Cauc.THC.Exposure	3.80 (3.52, 4.08)	<0.0001
Asian.THC.Exposure	0.61 (0.56, 0.65)	<0.0001
Hispanic	0.37 (0.29, 0.44)	<0.0001
Analgesics	0.27 (0.21, 0.33)	<0.0001
Alcohol.Abuse: THC_Exposure	202.56 (149.25, 255.88)	<0.0001
Alcohol.Abuse: THC_Exposure: Cannabigerol_Exposure	166.89 (122.33, 211.45)	<0.0001
Cigarettes: THC_Exposure: Cannabigerol_Exposure	40.72 (27.49, 53.96)	<0.0001
Cigarettes	338.49 (195.48, 481.49)	<0.0001
Cigarettes: Cannabigerol_Exposure	104.46 (60.12, 148.79)	<0.0001
Alcohol.Abuse: Cannabigerol_Exposure	269.86 (139.01, 400.71)	0.0001
Alcohol.Abuse	861.93 (440.79, 1283.06)	0.0001
Cannabigerol_Exposure	-17.40 (-27.04, -7.76)	0.0004
THC_Exposure: Cannabigerol_Exposure	-6.82 (-9.72, -3.92)	<0.0001
Median.HH.Income	-0.18 (-0.26, -0.10)	<0.0001
NH_Asian.Am	-0.23 (-0.31, -0.14)	<0.0001
Cigarettes: Alcohol.Abuse	-5236.74 (-7159.24, -3314.24)	<0.0001
Cigarettes: Alcohol.Abuse: Cannabigerol_Exposure	-1626.26 (-2222.8, -1029.72)	<0.0001
Cigarettes: Alcohol.Abuse: THC_Exposure	-1058.45 (-1299.49, -817.41)	<0.0001
Cigarettes: Alcohol.Abuse: THC_Exposure: Cannabigerol_Exposure	-919.50 (-1122.17, -716.83)	<0.0001
Afric.THC.Exposure	-3.13 (-3.34, -2.91)	<0.0001
**Model Interactive in Drugs & in Ethnic THC_Exposure**		
lme(Downs_Rate ~ Cigarettes*Alcohol.Abuse* THC.Exposure*Cannabigerol.Exposure*Analgesics+Cocaine+ Median.HH.Income+Cauc.Am.THC.Exposure*Afric.Am.THC.Exposure* Asian.Am.THCExposure+Hispanic.THC.Exposure+AIAN.THC.Exposure, random=~1|id, weights = ~ sw)		
Cigarettes: THC_Exposure: Cannabigerol_Exposure	18.82 (16.82, 20.82)	<0.0001
Alcohol.Abuse: THC_Exposure: Cannabigerol_Exposure	153.80 (134.19, 173.42)	<0.0001
Cannabigerol_Exposure	2.80 (2.39, 3.21)	<0.0001

Alcohol.Abuse: THC_Exposure	381.38 (315.58, 447.17)	<0.0001
Hispanic	0.46 (0.36, 0.57)	<0.0001
NH_Cauc.Am	1.88 (1.39, 2.38)	<0.0001
Cocaine	0.14 (0.1, 0.17)	<0.0001
Cigarettes: Cannabigerol_Exposure	14.74 (8.88, 20.60)	<0.0001
Cauc.THC.Exposure	11.09 (5.16, 17.02)	0.0003
Cigarettes	34.81 (13.74, 55.88)	0.0013
Cauc.THC.Exposure: Asian.THC.Exposure	5.47 (1.71, 9.22)	0.0045
Analgesics	-0.08 (-0.15, -0.01)	0.0304
Asian.THC.Exposure	-2.28 (-4.25, -0.31)	0.0238
Afric.THC.Exposure: Asian.THC.Exposure	-4.52 (-7.77, -1.28)	0.0065
Afric.THC.Exposure	-9.18 (-14.21, -4.15)	0.0004
Cauc.THC.Exposure: Afric.THC.Exposure	-0.51 (-0.7, -0.31)	<0.0001
THC_Exposure	-9.67 (-12.89, -6.44)	<0.0001
Cauc.THC.Exposure: Afric.THC.Exposure: Asian.THC.Exposure	-0.25 (-0.32, -0.17)	<0.0001
Cigarettes: Alcohol.Abuse	-1018.79 (-1301.96, -735.63)	<0.0001
Cigarettes: Alcohol.Abuse: Cannabigerol_Exposure	-375.33 (-455.41, -295.25)	<0.0001
THC_Exposure: Cannabigerol_Exposure	-5.11 (-6.18, -4.03)	<0.0001
Alcohol.Abuse	-52.69 (-63.1, -42.28)	<0.0001
Cigarettes: Alcohol.Abuse: THC_Exposure	-1204.10 (-1434.36, -973.83)	<0.0001
Cigarettes: Alcohol.Abuse: THC_Exposure: Cannabigerol_Exposure	-639.23 (-709.47, -568.99)	<0.0001

Abbreviations: iid - independent identically distributed, 5 Races – NH Caucasian-American, NH African American, Hispanic, Asian American and American Indian, Alaska Native.

Supplementary Table S8. : Linear Regression of Mulitvariable Adjustment for Cannabis Legal Status.

Model and Term	Term Parameters		Model Parameters	
	Estimate (C.I.)	P-Value	Parameter	Value
**Legal Status**				
Additive Cannabinoids				
lm(Downs_Rate ~ Cigarettes+Alcohol.Abuse+THC.Exposure+Cannabigerol.Exposure+Analgesics+Cocaine+4_Races+ Median.HH.Income+Status, weights = sw)				
sNHWhite + asinh(sNHBlack) + asinh(sHispanic) + asinh(sNHAsian) + asinh(sMHY) + Status				
Median Household Income	0.87 (0.665, 1.07)	8.31E-16	Adj R Squ.	0.626
Status - Decriminalised	0.39 (0.176, 0.611)	4.18E-04	F	46.12
Status - Medical	0.38 (0.0659, 0.697)	1.80E-02	dF	13, 337
THC.Exposure	0.37 (0.08, 0.664)	1.27E-02	S.D.	0.0473
NHBlack	0.34 (0.211, 0.458)	1.65E-07	P	9.35E-67
Analgesics	0.27 (0.134, 0.403)	1.03E-04		
Alcoholism	0.26 (0.157, 0.359)	8.25E-07		
Hispanic	0.24 (0.0816, 0.398)	3.07E-03		
Cigarettes	0.21 (0.061, 0.348)	5.37E-03		
Cocaine	-0.3 (-0.445, -0.149)	9.69E-05		
Asian	-0.48 (-0.84, -0.125)	8.24E-03		
CBG.Exposure	-0.49 (-0.823, -0.16)	3.79E-03		
Status - Legal	-1.05 (-75.8, 73.7)	9.78E-01		

Interactive Cannabinoids				
*lm(Downs_Rate ~ Cigarettes*Alcohol.Abuse*THC.Exposure*Cannabigerol.Exposure+Analgesics+Cocaine+4_Races+ Median.HH.Income+Status, weights = sw)*				
NHWhite: NHBlack: Hispanic: Asian	43.7 (37.9, 49.5)	3.22E-38	Adj R Squ.	0.8601
NHBlack: Hispanic: Asian	36.7 (32.3, 41)	2.45E-45	F	77.87
NHWhite: NHBlack: Asian	21.9 (18.6, 25.2)	3.04E-31	dF	28, 322
NHWhite: Hispanic: Asian	21.9 (18.4, 25.4)	1.10E-28	S.D.	0.0289
NHBlack: Asian	18.8 (16, 21.5)	3.03E-33	P	9.74E-126
Hispanic: Asian	15 (12.2, 17.8)	1.23E-22		
NHWhite: Asian	13.7 (11.4, 16)	1.18E-26		
NHWhite: NHBlack: Hispanic	11.9 (10.3, 13.6)	2.56E-35		
NHBlack: Hispanic	11.1 (9.79, 12.5)	1.14E-43		
Asian	8.96 (7.44, 10.5)	4.56E-26		
NHBlack	5.43 (4.67, 6.19)	3.03E-35		
NHWhite: NHBlack	4.97 (4.09, 5.85)	1.52E-24		
NHWhite: Hispanic	4.33 (3.52, 5.15)	3.92E-22		
Hispanic	3.64 (2.94, 4.35)	4.03E-21		
NHWhite	2.61 (2.15, 3.07)	9.54E-25		
Status - Medical	2.47 (2.13, 2.81)	2.00E-36		
CBG.Exposure	0.65 (0.414, 0.886)	1.17E-07		
Cigarettes: CBG.Exposure	0.52 (0.052, 0.994)	2.96E-02		
Analgesics	0.5 (0.373, 0.618)	3.07E-14		
Status - Decriminalised	0.42 (0.263, 0.577)	2.60E-07		
Cigarettes: THC.Exposure: CBG.Exposure	0.39 (0.165, 0.609)	6.77E-04		
Median Household Income	0.29 (0.0792, 0.491)	6.80E-03		
Cigarettes	-0.26 (-0.489, -0.0392)	2.15E-02		
Cigarettes: THC.Exposure	-0.39 (-0.795, 0.0124)	5.74E-02		
Cocaine	-0.4 (-0.556, -0.249)	4.57E-07		
Cigarettes: Alcoholism	-0.41 (-0.486, -0.33)	1.15E-21		
Alcoholism: CBG.Exposure	-0.95 (-1.07, -0.819)	5.40E-38		
Status - Legal	-1.65 (-47.4, 44.1)	9.44E-01		
**Dichotomous Legal Status**				
Additive Cannabinoids				
lm(Downs_Rate ~ Cigarettes+Alcohol.Abuse+THC.Exposure+Cannabigerol.Exposure+Analgesics+Cocaine+4_Races+ Median.HH.Income+ Dichotomized.Status, weights = sw)				
Median Household Income	0.87 (0.666, 1.07)	6.37E-16	Adj R Squ.	0.6285
Dichotomous Status - Liberal	0.39 (0.212, 0.567)	2.05E-05	F	54.83
THC.Exposure	0.38 (0.0965, 0.653)	8.45E-03	dF	11, 339
NHBlack	0.33 (0.212, 0.457)	1.52E-07	S.D.	0.0472
Analgesics	0.27 (0.149, 0.383)	1.06E-05	P	1.74E-68
Alcoholism	0.26 (0.158, 0.357)	5.52E-07		
Hispanic	0.24 (0.0851, 0.392)	2.42E-03		
Cigarettes	0.21 (0.0624, 0.348)	4.98E-03		
Cocaine	-0.3 (-0.435, -0.156)	3.99E-05		
Asian	-0.49 (-0.827, -0.144)	5.48E-03		

CBG.Exposure	-0.49 (-0.813, -0.174)	2.55E-03		

Interactive Cannabinoids				
*lm(Downs_Rate ~ Cigarettes*Alcohol.Abuse*THC.Exposure*Cannabigerol.Exposure+Analgesics+Cocaine+4_Races+ Median.HH.Income+ Dichotomized.Status, weights = sw)*				
NHWhite: NHBlack: Hispanic: Asian	42.2 (35.6, 48.7)	2.13E-30	Adj R Squ.	0.8216
NHBlack: Hispanic: Asian	33 (28.2, 37.8)	1.30E-33	F	65.45
NHWhite: Hispanic: Asian	22.7 (18.9, 26.6)	2.01E-26	dF	25, 325
NHWhite: NHBlack: Asian	21.8 (18, 25.5)	3.81E-25	S.D.	0.0327
NHBlack: Asian	17.7 (14.7, 20.8)	3.65E-25	P	5.23E-111
Hispanic: Asian	16.2 (13.3, 19.2)	2.47E-23		
NHWhite: Asian	13.7 (11.1, 16.3)	6.83E-22		
NHWhite: NHBlack: Hispanic	11.4 (9.52, 13.3)	1.80E-27		
NHBlack: Hispanic	9.88 (8.39, 11.4)	9.79E-32		
Asian	8.24 (6.55, 9.93)	2.74E-19		
NHBlack	5.16 (4.31, 6.01)	1.77E-27		
NHWhite: NHBlack	4.88 (3.89, 5.86)	7.00E-20		
NHWhite: Hispanic	4.74 (3.83, 5.64)	1.01E-21		
Hispanic	4.45 (3.68, 5.23)	3.50E-25		
NHWhite	2.54 (2.01, 3.06)	5.10E-19		
Cigarettes: Alcoholism: THC.Exposure	1.13 (0.864, 1.39)	1.17E-15		
CBG.Exposure	0.96 (0.703, 1.21)	7.96E-13		
Dichotomous Status - Liberal	0.82 (0.663, 0.974)	6.20E-22		
Median Household Income	0.69 (0.495, 0.89)	2.77E-11		
Analgesics	0.61 (0.482, 0.746)	5.19E-18		
Alcoholism: THC.Exposure	-0.26 (-0.505, -0.00826)	4.29E-02		
Cigarettes: CBG.Exposure	-0.26 (-0.435, -0.0837)	3.93E-03		
Alcoholism: CBG.Exposure	-0.52 (-0.808, -0.238)	3.51E-04		
Cocaine	-0.66 (-0.778, -0.539)	1.49E-23		
Cigarettes: Alcoholism: CBG.Exposure	-1.13 (-1.51, -0.746)	1.34E-08		

Dependent Variable is the Down Syndrome rate.

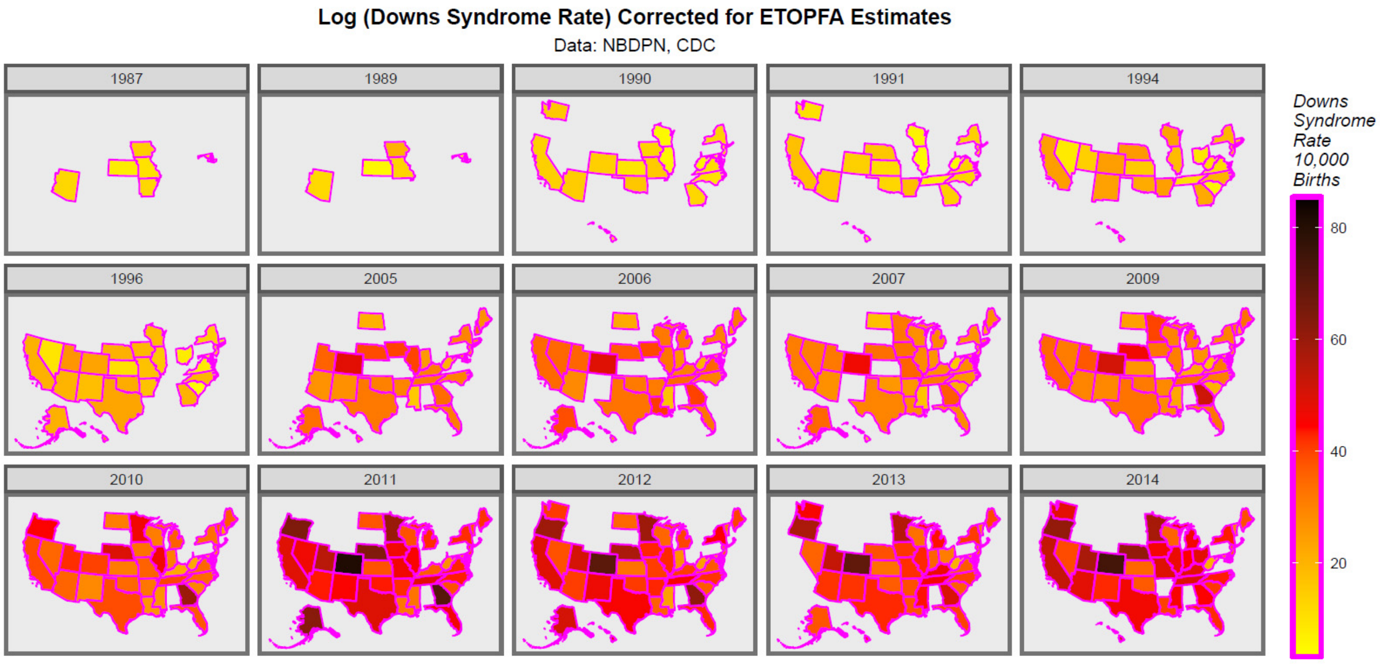

Supplementary Figure S1. Map graph of the logarithm of the Down syndrome rate corrected for estimates of the early termination of pregnancy for anomaly (ETOPFA) rate.

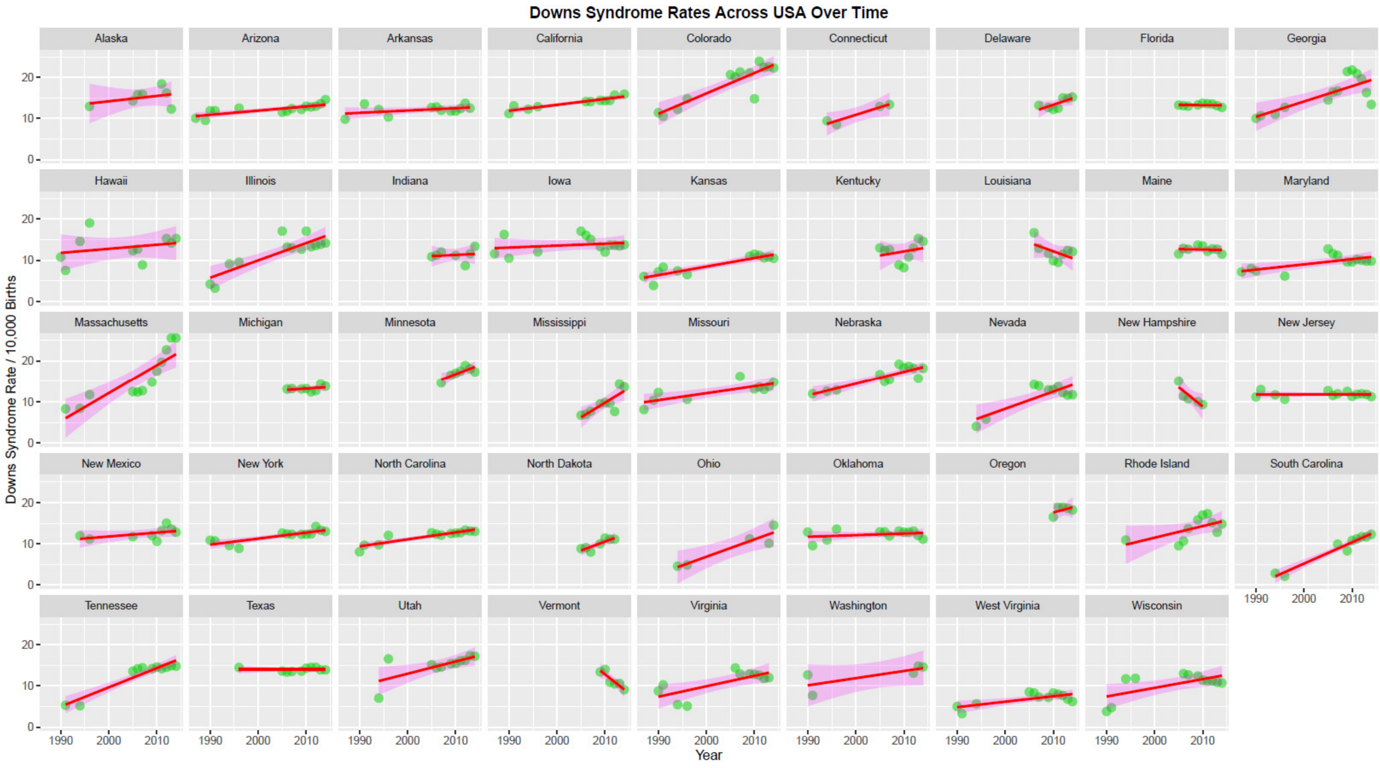

Supplementary Figure S2. Down syndrome rate across USA over time as a panelled and facetted scatterplot in alphabetical order.

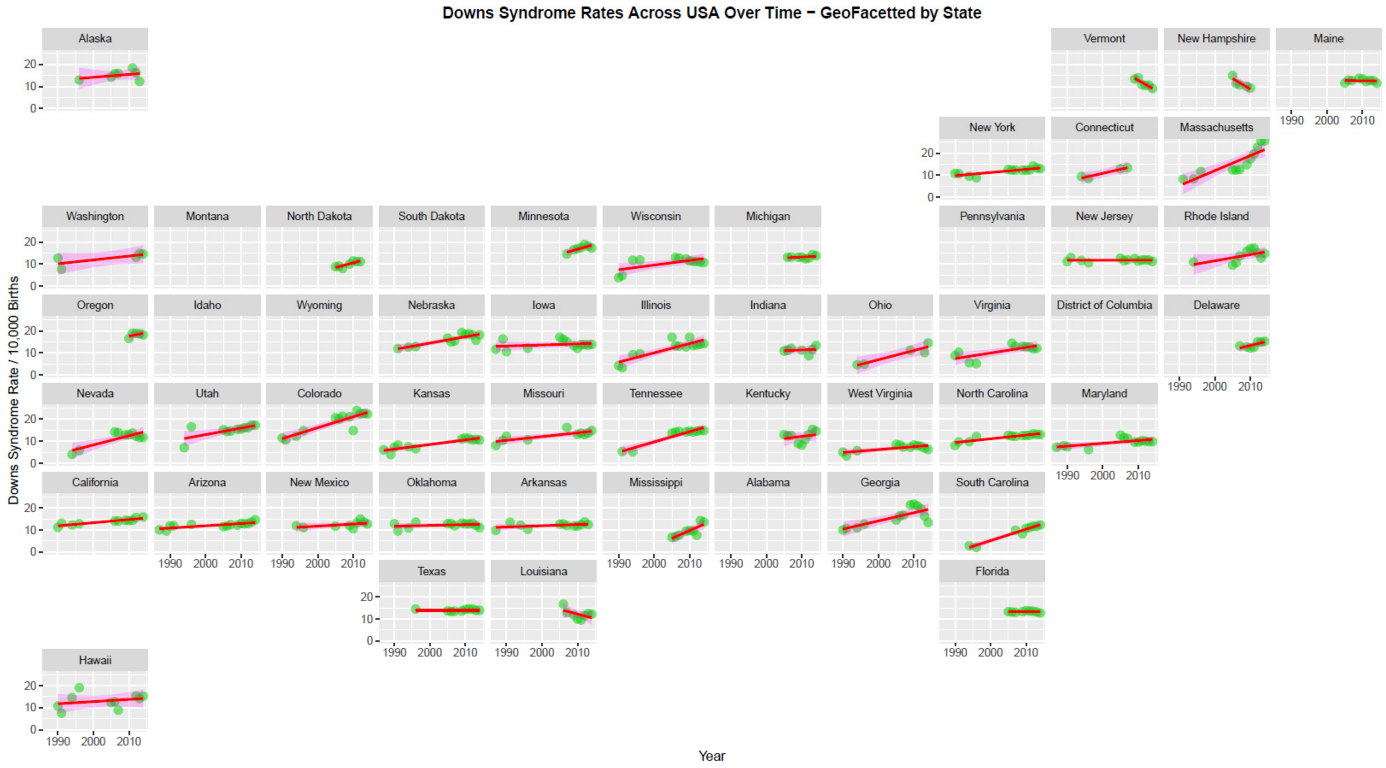

Supplementary Figure S3. Down syndrome rate across USA over time as a geospatial panelled geofacetted scatterplot with each state in approximately its correct spatial location.

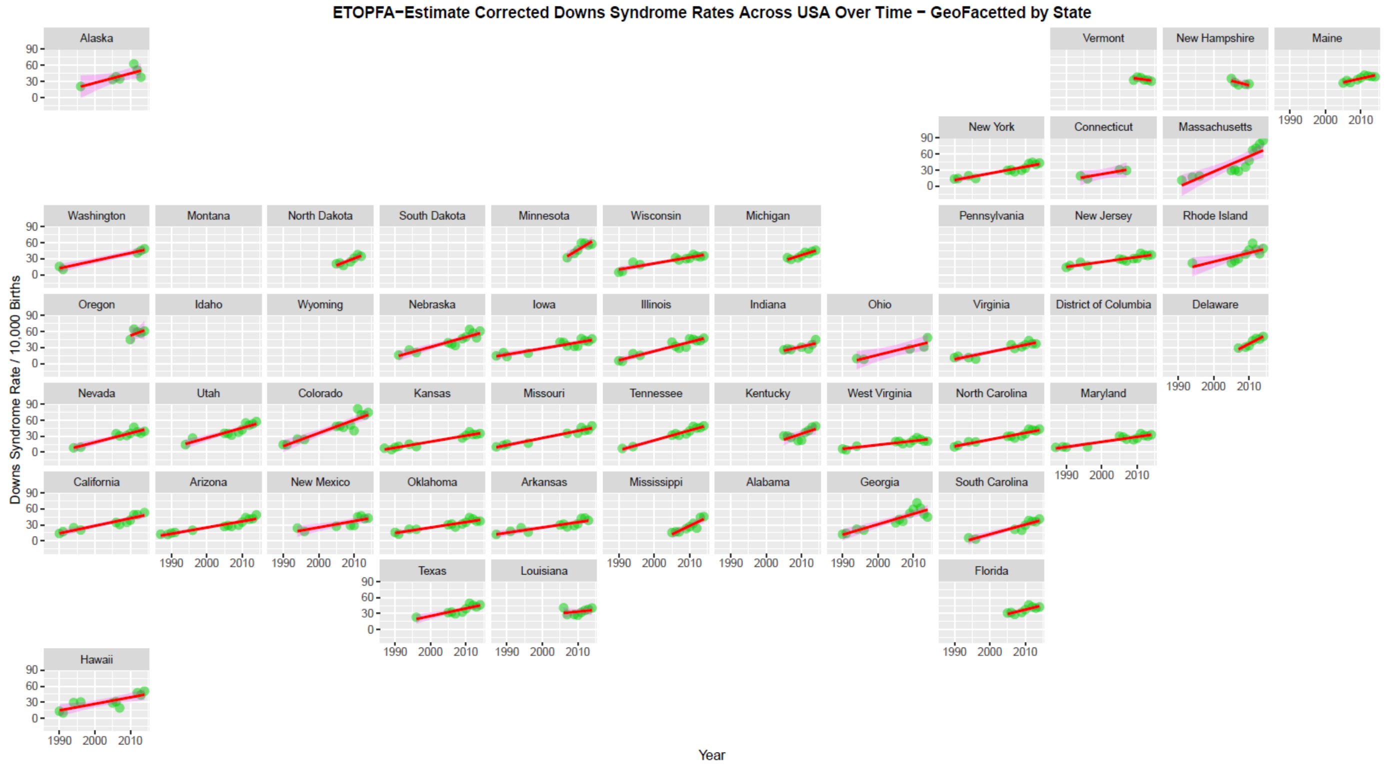

Supplementary Figure S4. ETOPFA-corrected Down syndrome rate across USA over time as a geospatial panelled geofacetted scatterplot with each state in approximately its correct spatial location.

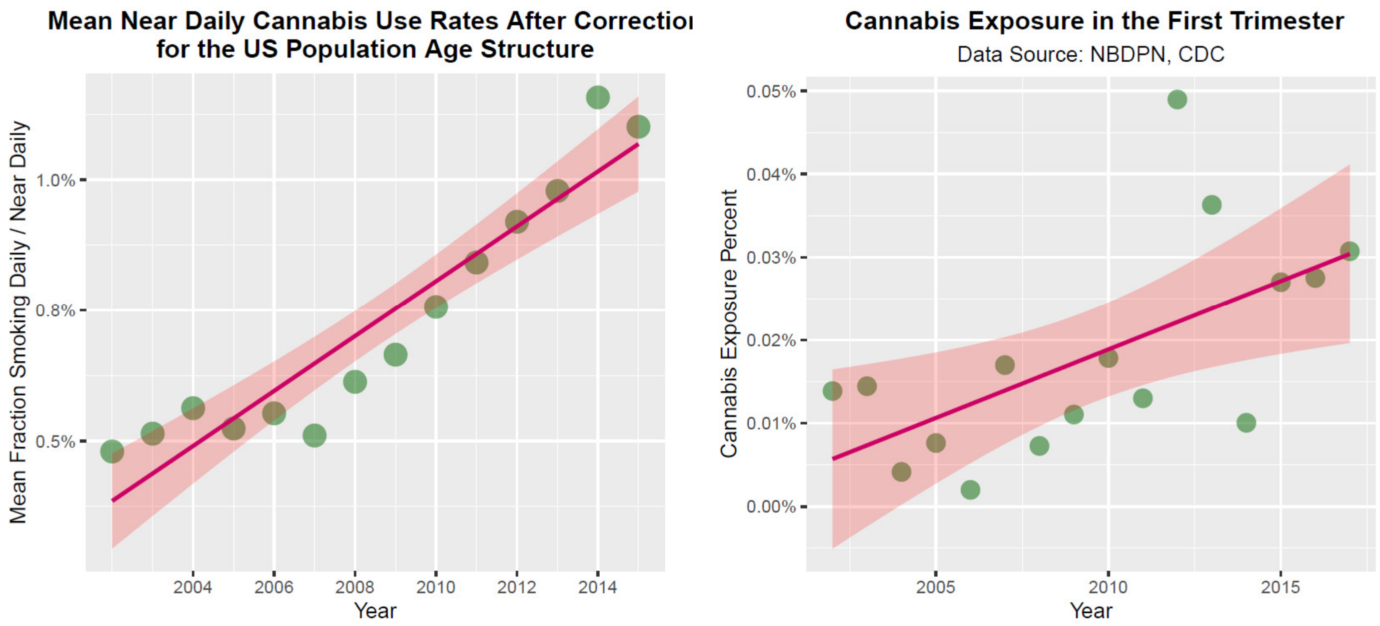

Supplementary Figure S5. (A) Near daily cannabis use in pregnancy in NSDUH survey and (B) cannabis exposure in first trimester.

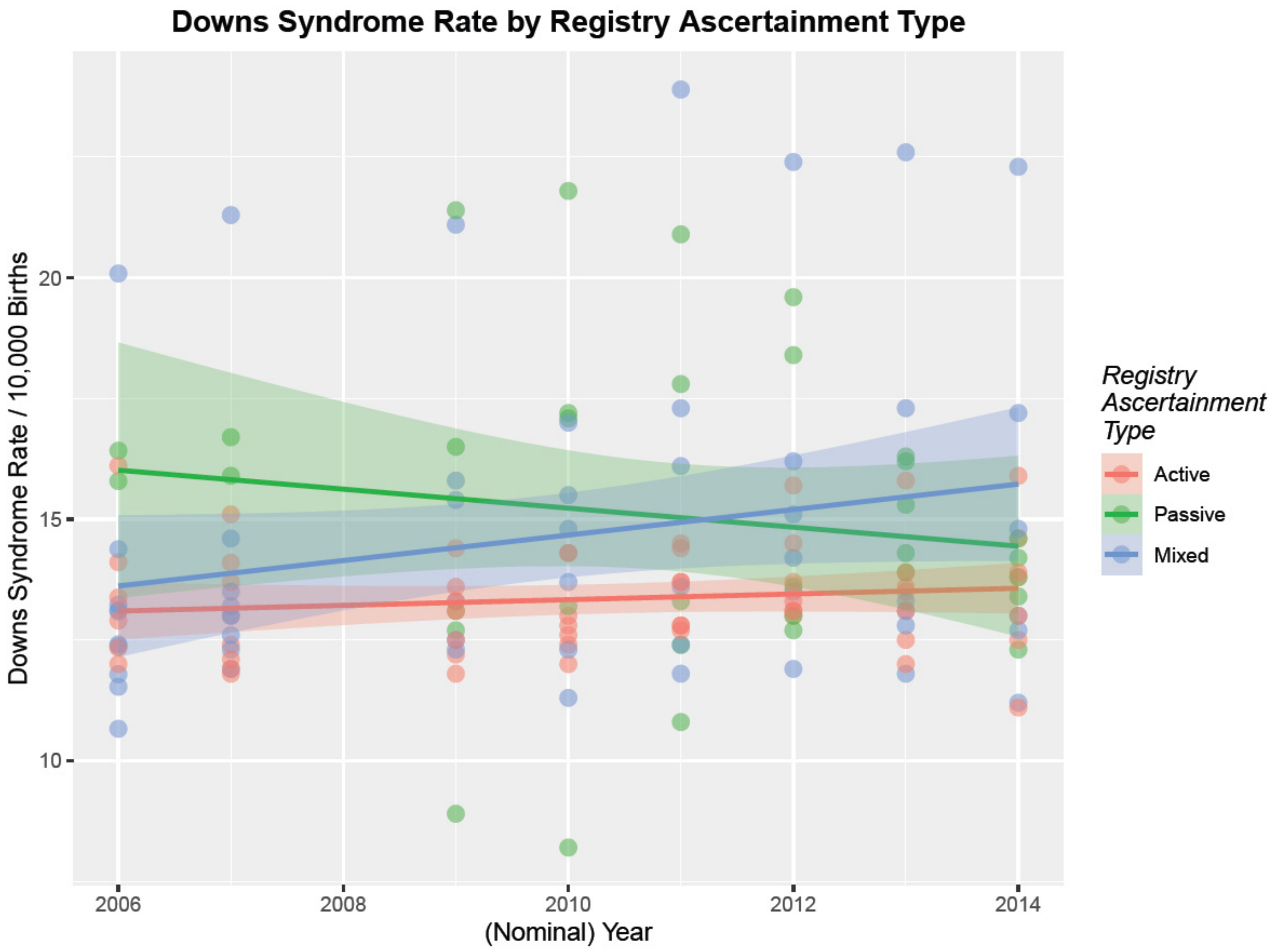

Supplementary Figure S6. Scatterplot of the effect of registry case ascertainment on the reported Down syndrome case rate.

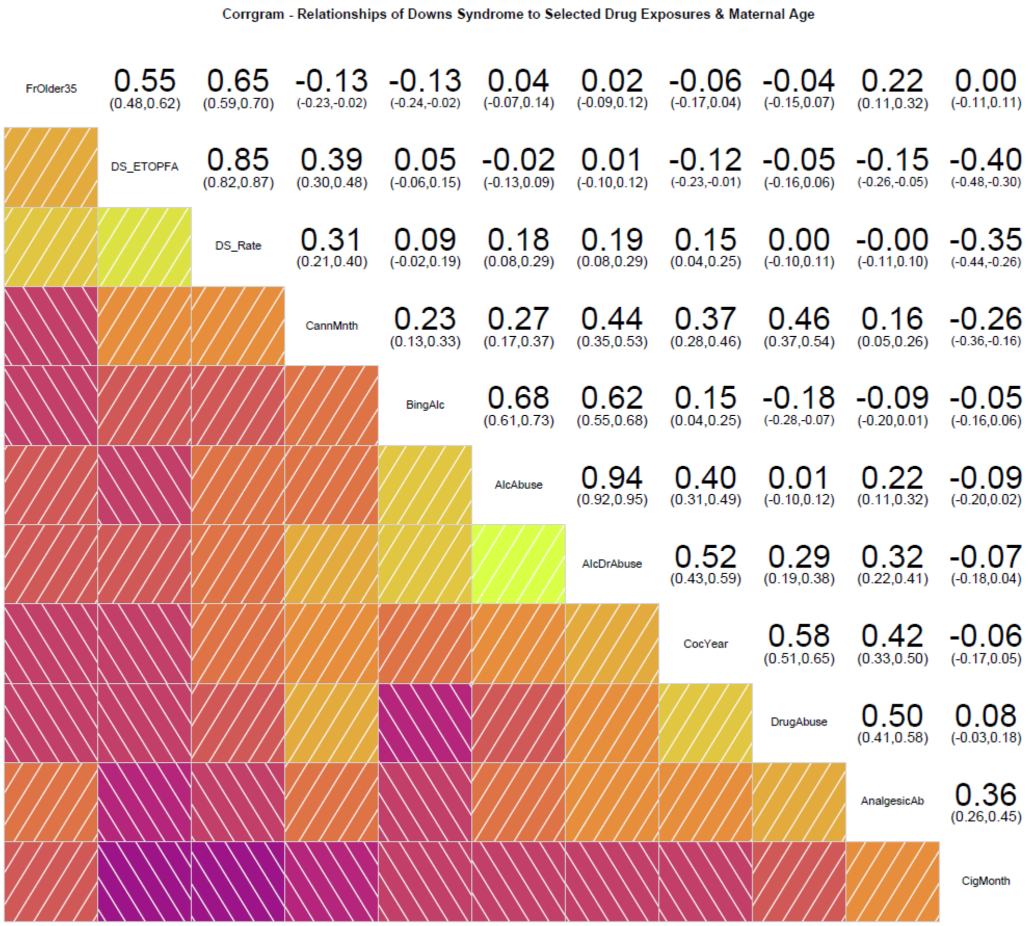

Supplementary Figure S7. Corrgram correlogram of the Down syndrome rate by selected drug exposures and maternal ages.

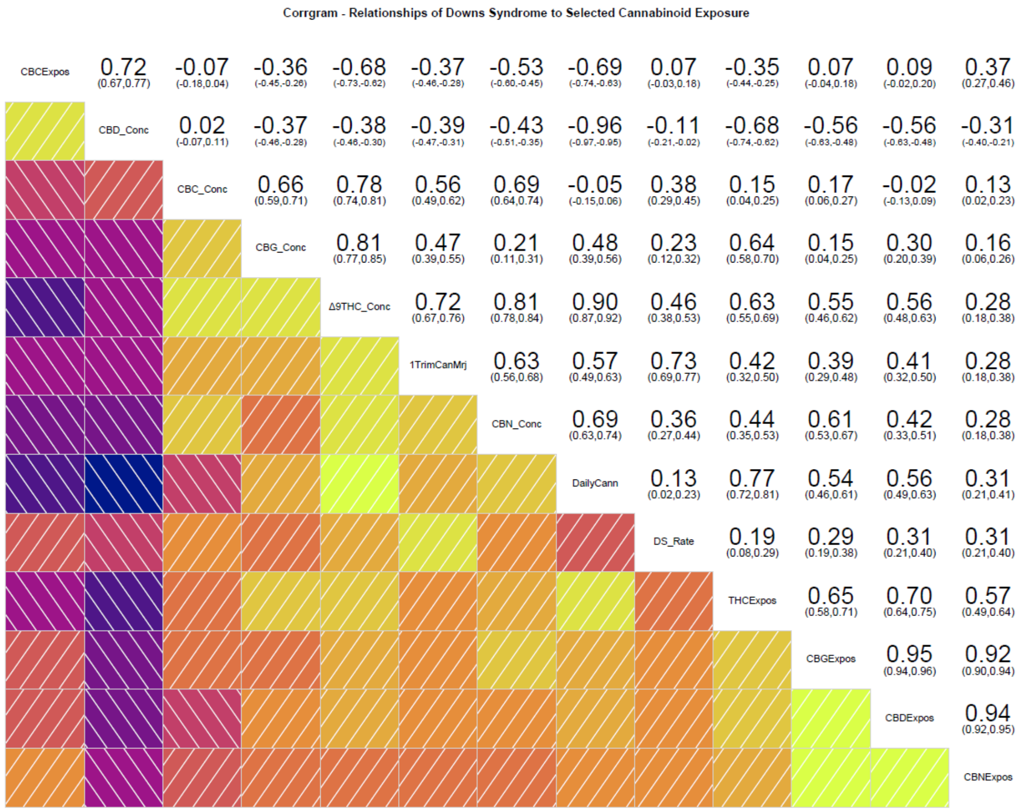

Supplementary Figure S8. Corrgram correlogram of the Down syndrome rate by estimates of selected cannabinoid exposures.

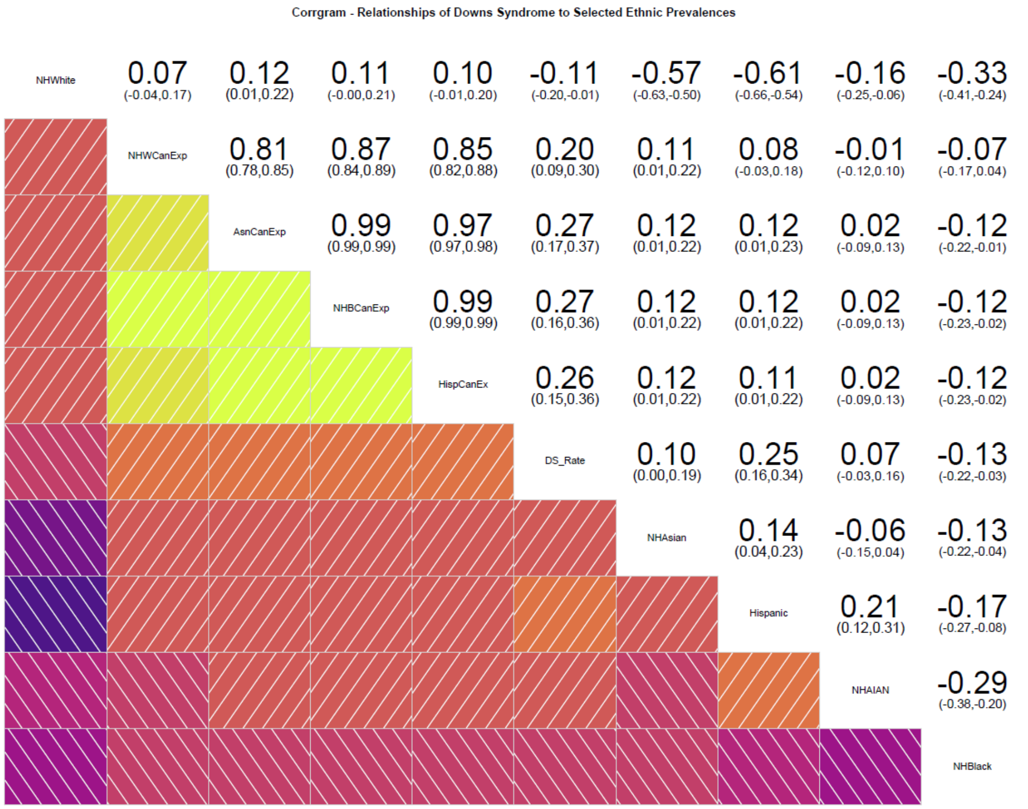

Supplementary Figure S9. Corrgram correlogram of the Down syndrome rate by selected ethnic prevalences.

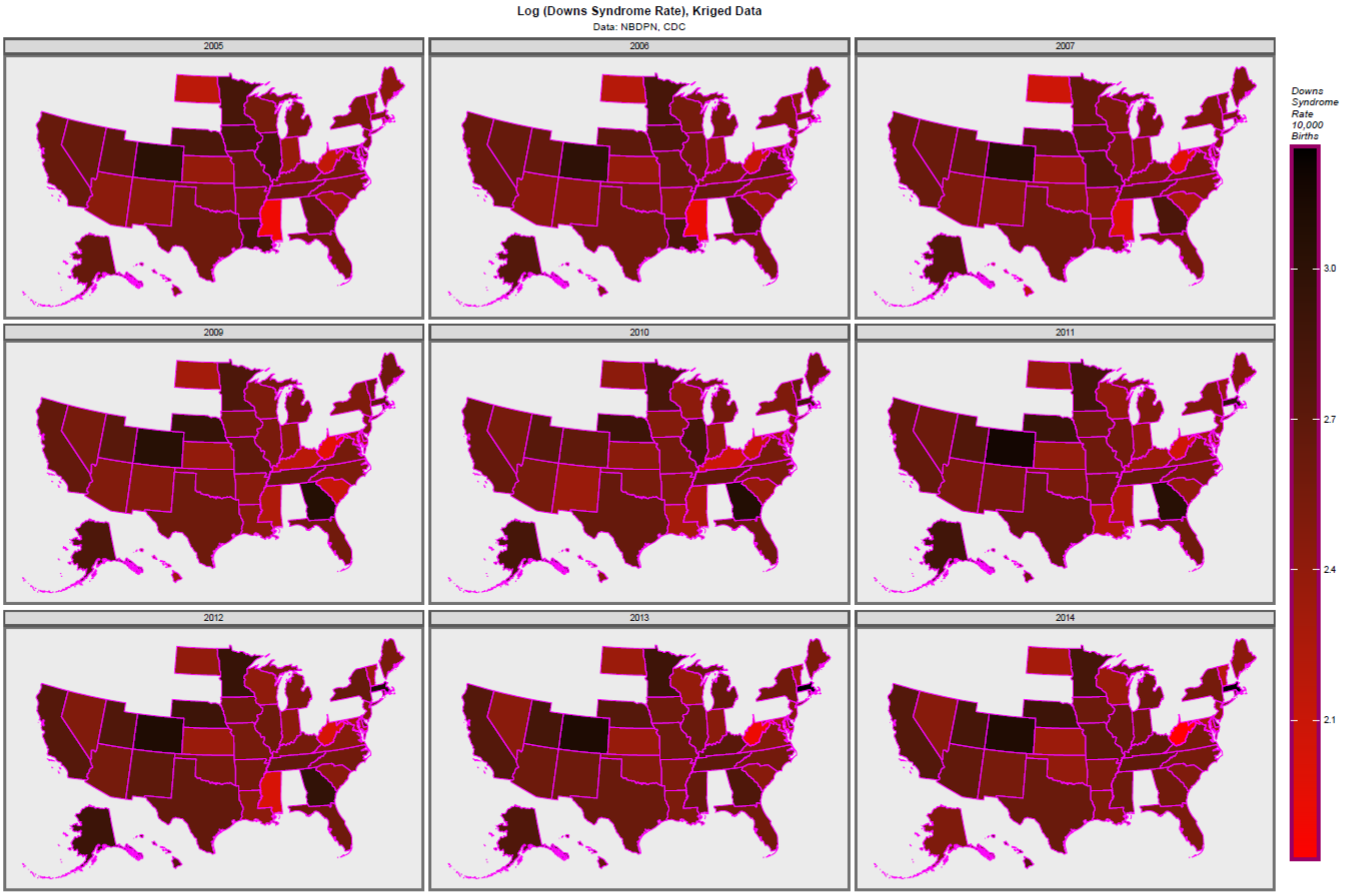

Supplementary Figure S10. Map graph of the logarithm of the kriged Down syndrome rate.

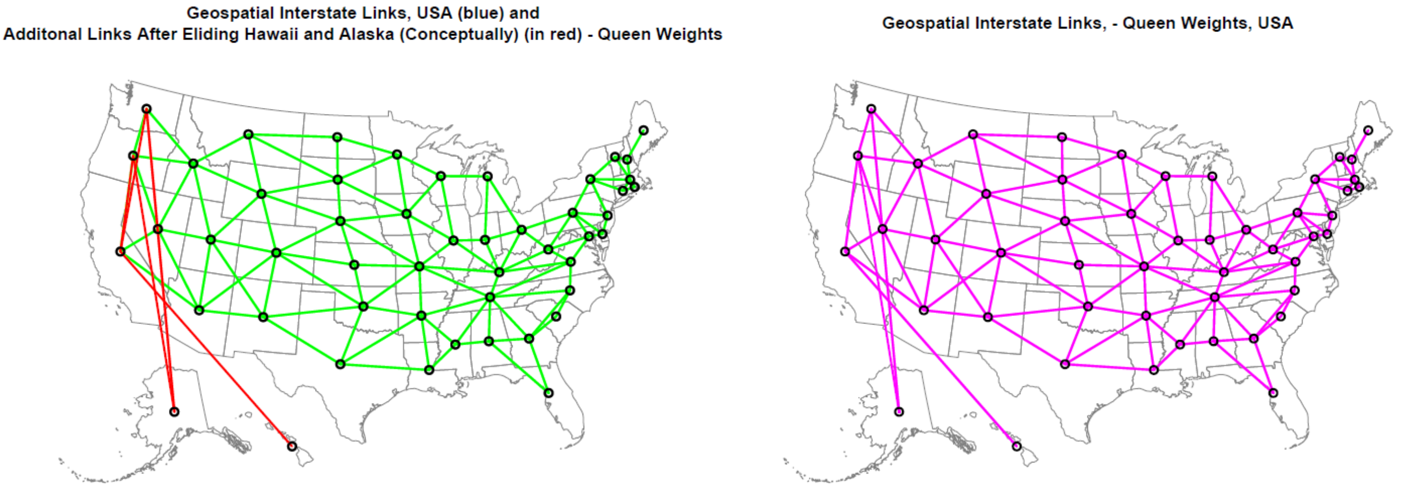

Supplementary Figure S11. (A) edited and (B) final interstate geospatial links which comprise the sparse spatial weights matrix used in all geospatial regressions.
